# Breast cancer exosomes contribute to pre-metastatic niche formation and promote bone metastasis of tumor cells

**DOI:** 10.7150/thno.45351

**Published:** 2021-01-01

**Authors:** Xinxin Yuan, Niansong Qian, Shukuan Ling, Yuheng Li, Weijia Sun, Jianwei Li, Ruikai Du, Guohui Zhong, Caizhi Liu, Guotao Yu, Dengchao Cao, Zizhong Liu, Yinbo Wang, Zhihong Qi, Yingpeng Yao, Fang Wang, Jingjing Liu, Shanshan Hao, Xiaoyan Jin, Yinlong Zhao, Jianqi Xue, Dingsheng Zhao, Xingcheng Gao, Shuai Liang, Youyou Li, Jinping Song, Shuyang Yu, Yingxian Li

**Affiliations:** 1 State Key Laboratory of Agrobiotechnology, College of Biological Sciences, China Agricultural University, Beijing 100193, China.; 2 State Key Lab of Space Medicine Fundamentals and Application, China Astronaut Research and Training Center, Beijing 100094, China.; 3 Department of Oncology, Chinese PLA General Hospital, Beijing 100853, China.

**Keywords:** exosomes, pre-metastatic niche, osteoclasts, miR-21, bone metastasis

## Abstract

**Rationale:** Breast cancer preferentially develops osteolytic bone metastasis, which makes patients suffer from pain, fractures and spinal cord compression. Accumulating evidences have shown that exosomes play an irreplaceable role in pre-metastatic niche formation as a communication messenger. However, the function of exosomes secreted by breast cancer cells remains incompletely understood in bone metastasis of breast cancer.

**Methods:** Mouse xenograft models and intravenous injection of exosomes were applied for analyzing the role of breast cancer cell-derived exosomes *in vivo*. Effects of exosomes secreted by the mildly metastatic MDA231 and its subline SCP28 with highly metastatic ability on osteoclasts formation were confirmed by TRAP staining, ELISA, microcomputed tomography, histomorphometric analyses, and pit formation assay. The candidate exosomal miRNAs for promoting osteoclastogenesis were globally screened by RNA-seq. qRT-PCR, western blot, confocal microscopy, and RNA interfering were performed to validate the function of exosomal miRNA.

**Results:** Implantation of SCP28 tumor cells *in situ* leads to increased osteoclast activity and reduced bone density, which contributes to the formation of pre-metastatic niche for tumor cells. We found SCP28 cells-secreted exosomes are critical factors in promoting osteoclast differentiation and activation, which consequently accelerates bone lesion to reconstruct microenvironment for bone metastasis. Mechanistically, exosomal miR-21 derived from SCP28 cells facilitates osteoclastogenesis through regulating PDCD4 protein levels. Moreover, miR-21 level in serum exosomes of breast cancer patients with bone metastasis is significantly higher than that in other subpopulations.

**Conclusion:** Our results indicate that breast cancer cell-derived exosomes play an important role in promoting breast cancer bone metastasis, which is associated with the formation of pre-metastatic niche via transferring miR-21 to osteoclasts. The data from patient samples further reflect the significance of miR-21 as a potential target for clinical diagnosis and treatment of breast cancer bone metastasis.

## Introduction

Breast cancer is the second leading cause of cancer-related mortality among women, and bone metastasis is frequently observed in breast cancer patients during the disease progression 1. Osteolytic bone metastasis occurs in 80% of advanced breast cancer patients, accompanied by a variety of debilitating skeletal complications such as nerve compression, pathological fractures and hypercalcemia 2, 3. Traditional treatments for breast cancer patients including radiotherapy, chemotherapy, and adjuvant chemotherapy, even the recent approval of several bone-specific agents only alleviate skeletal-related complications, but mortality rates keep a high level due to acquisition of treatment resistance 4. Bone metastasis of breast cancer remains the most common threat to breast cancer patients, therefore further studies are needed to delineate the detailed mechanism for bone metastasis of breast cancer and discover new potential biomarkers for clinical diagnosis and treatment.

Over the past decades, accumulating evidence has demonstrated that breast cancer cells with bone metastatic tropism show specific intrinsic molecular characteristics 5, 6. Meanwhile, primary tumor stroma and specific stromal components in distant organs also dictate bone metastasis of breast cancer, such as interactions between tumor and vasculature that contribute to metastatic preference for bone tissue 7, 8. Bone is a dynamic tissue as one of the most common sites for cancer metastasis, and undergoes life-span rebuilding regulated by the bone-resorbing osteoclasts and bone-forming osteoblasts 9. In addition to the unique physiochemical properties of the bone environment, the cellular architecture of the bone marrow niche, including osteoclast, osteoblast, and hematopoietic stem cell niches which play fundamental roles in bone development, remodeling, and repair, provides advantages for metastatic colonization of breast cancer 10, 11. Recent studies reveal that breast cancer cells secrete a large panel of molecules, such as vascular cell adhesion molecule 1, intercellular adhesion molecule 1, lysyl oxidase, and IL-6, which directly or indirectly stimulate osteoclast activity 12. These extracellular communications facilitate bone degradation and shape the pre-metastatic niche to promote breast cancer progression, suggesting forming a feedback loop of bone metastasis 13, 14.

Exosomes are extracellular membrane vesicles with a diameter ranging from 30 to 200 nm, which mediate intercellular communication via transferring a wealth of nucleic acids and protein among cells under physiological and pathological conditions 15-17. Recently, accumulating studies demonstrate that tumor-derived exosomes contribute to generating suitable microenvironments for engraftment and colonization of incoming metastatic tumor cells 18-20. Prostate cancer-secreted exosomal hsa-miR-940 and pyruvate kinase M2 induce pre-metastatic osteoblastic lesions by promoting osteogenic differentiation of host mesenchymal cells 21, 22. Breast cancer-derived exosomes can destroy vascular endothelial barriers in distant organs to increase the incidence of brain and lung metastasis 23, 24. However, whether breast cancer-secreted exosomes influence the osteolytic bone metastasis through formation of pre-metastatic microenvironment remains largely unclear. Identification of these mechanisms may reveal new avenues for developing novel therapeutic targets for breast cancer.

Recent evidence suggests that breast cancer-derived miRNAs play key roles in tumor development and progression via exosomes transfer, including regulating the outgrowth and metastasis of breast tumor 25, 26 and microenvironment of metastatic sites 27, 28. miR-21, a highly conserved oncomicroRNA, is expressed in serum of many types of cancer patients, including hepatocellular carcinoma, lung cancer, and breast cancer, which is significantly higher than that in healthy controls 29-31. Several studies demonstrate that miR-21 involves in tumor progression and osteoclasts differentiation 32, 33. For example, miR-21 promotes NFATc1 up-regulation during osteoclast differentiation by directly binding programmed cell death 4 (PDCD4), which has a suppressive function on c-Fos transactivation 34. However, whether the breast cancer-derived exosomal miR-21 corresponds to osteoclast-driven pre-metastatic niche for bone metastasis of breast tumor is waiting to be determined.

In this study, we found breast cancer cell-secreted exosomes contributed to the generation of pre-metastatic niche by promoting osteoclast differentiation and enhancing bone metastasis. Mechanically, breast cancer-derived exosomal miR-21 decreased the expression of PDCD4. Importantly, the expression level of miR-21 was detected at higher level in serum exosomes of breast cancer patients with bone-metastasis than that in patients without bone-metastasis. Our findings uncover a previously unknown mechanism on bone-metastasis of breast cancer, providing new insights on clinical diagnosis and treatment.

## Materials and Methods

### Animal experiments

All mice were bred and housed in specific pathogen-free conditions under controlled temperature (22 ± 1 °C) and exposed to a constant 12 h light-dark cycle in the animal facilities at China Astronaut Research and Training Center. 6 to 8-week-old BALB/c nude female mice (Charles river, China) were used for the overall experiments in this study. Animal experiments were conducted in accordance with the guidelines and with the approval of the Committees of Animal Ethics and Experimental Safety of China Astronaut Research and Training Center.

For the generation of models with orthotopic primary tumor, female BALB/c nude mice at 6 to 8-week-old were anaesthetized and a small incision was made to expose the mammary gland. 10^5^ luciferase-labeled SCP28 cells suspended in 10 µl PBS were injected directly into the fat pad. For bone metastasis assay, 2 × 10^5^ SCP28 or MDA-MB-231 cells suspended in 100 μL PBS were injected into the caudal artery of anesthetized mice by 29 G syringe needle. For bisphosphonate assay, 0.6 mg/kg zoledronic acid was injected intraperitoneally twice a week.

Tumor burden was monitored by measuring photon flux of bioluminescent imaging (BLI) signals using IVIS Spectrum CT (PerkinElmer) after intraperitoneal injection of 150 mg/kg D-Luciferin (PerkinElmer, USA). Data were normalized to the signal on day 0. Bone damages were measured by X-ray radiography with a Faxitron instrument (Faxitron X-ray, USA) and quantified by ImageJ software 1.52 (NIH, Bethesda, MD, USA). At the checkpoint, bone samples were used for bone histology analysis and bone density quantification by Micro-computed tomography (Micro-CT) imaging.

### X-Ray imaging and osteolytic lesion quantification

The osteolytic status of the hind limbs was observed by X-ray photography. Anesthetized mice were placed in prone position and exposed to X-ray radiography at 45 kV for 2 s using a MX-20 Faxitron instrument. The osteolytic area in the hind limbs was quantified using the ImageJ software 1.52 (NIH, Bethesda, MD, USA).

### Bone histomorphometric analysis

The tibia of mice was fixed with 4% paraformaldehyde for 48 h followed by decalcification in 10% EDTA for 3-4 weeks, and 6 μm sections were prepared on a rotation microtome. Paraffin-embedded sections were deparaffinized in xylene and rehydrated. The samples were next performed hematoxylin-eosin (H&E) and tartrate resistant acid phosphatase (TRAP) staining (Sigma, USA) according to the standard protocol. For H&E staining, the bone sections from 4 mice per group were obtained, and 6 fields of view from total 4 slides per mouse were randomly selected for photography.

For immunohistochemical staining, paraffin-embedded sections were deparaffinized in xylene and rehydrated. Antigen retrieval was performed with protease K at 37 °C for 15 min. The activity of endogenous peroxidase was blocked with 3% H_2_O_2_ solution. The samples were stained overnight at 4 °C with GFP-tag mouse monoclonal antibody (1:100, AB0005, Abways) or rabbit polyclonal antibody against osteocalcin (OCN) (1:400, 23418-1-AP, Proteintech). After three times washing with PBS, biotinylated secondary antibodies were then added and incubated for 1 h at room temperature. Negative-control experiments were carried out by omitting the primary antibodies. DAB (ZSGB-bio, China) was used as chromogen, and hematoxylin was used to counterstain. The sections were examined using a microscope (ECLIPSE Ci-S, Nikon, Japan). Statistical analyses were performed with the Osteomeasure Analysis System, including the following parameters: osteoclast surface/bone surface (Oc.S/BS), osteoclast number/bone perimeter (N.Oc/B.Pm), osteoblast surface/bone surface (Ob.S/BS) and osteoblast number/bone perimeter (N.Ob/B.Pm).

### Micro-CT analysis

High-resolution micro-CT analysis was performed on the distal femurs using a model of μ40 Scanco (SCANCO Medical, Bruttisellen, Switzerland). In the femurs, the trabecular bone proximal to the distal growth plate was selected for analysis within a conforming volume of interest (cortical bone excluded) commencing at a distance of 840 μm from the growth plate and extending a further longitudinal distance of 1680 μm in the proximal direction. Cortical measurements were performed in the diaphyseal region of the femur starting at a distance of 3.57 mm from the growth plate and extending a further longitudinal distance of 210 μm in the proximal direction. All trabecular bone from each selected slice was segmented for three-dimensional reconstruction to calculate the following parameters: trabecular bone mineral density (BMD), trabecular bone volume (BV/TV), trabecular thickness (Tb.Th), structure model index (SMI), trabecular number (Tb.N), trabecular separation (Tb.Sp) and cortical thickness (C.Th).

### ELISA

ELISA was conducted with collected murine and human serum using ELISA kits according to the manufacturers' instructions. Serological levels of cross-linked C-telopeptide of type I collagen (CTX-1) and I type collagen amino end before collagen peptide (PINP) were detected using ELISA Kits (KE1744, Immunoway, USA; AE90833Mu, AMEKO, China), respectively. Human serum samples were collected from 21 breast cancer patients without relapse, 9 patients with other sites of relapse and 21 breast cancer patients with bone metastasis in the Chinese PLA General Hospital. All procedures were approved by the Committees of Clinical Ethics in the Institute of the General Hospital of Chinese People's Liberation Army. Patient consent was obtained before the start of the study. The exosomal miR-21 in human serum was measured with exoRNeasy Serum/Plasma Midi Kit (1088400, QIAGEN).

### Cell sorting

The bone marrow cells and bone marrow stromal cells were collected from the femur and tibia of the control and exosomes-conditioned mice. After washed with PBS containing 1% BSA, the cells were directly stained with goat against mouse Oscar (1:40, Santa Cruz Biotechnology, USA) and then stained with donkey anti-goat IgG-PE (1:100, R&D systems, USA). Oscar positive cells were sorted with a FACSAria II (BD Biosciences, USA). All data were analyzed using FlowJo software v.10 (BD Biosciences, USA).

### Cell culture

SCP28 and MDA-MB-231 cells were cultured in full DMEM (supplemented with 10% FBS which was depleted of exosomes by ultracentrifugation at 120,000 g for 70 min, 1% penicillin, and 1% streptomycin). The bone marrow monocyte (BMM) cells were induced into osteoclasts in full α-MEM contained with 30 ng/mL recombinant human M-CSF (R&D systems, USA) and 50 ng/mL RANKL (R&D systems, USA). Cells were routinely maintained in a humidified incubator at 37 °C with 5% CO_2_ and the culture medium was replaced for fresh every 2 days. To collect the exosomes from distinct cultural conditions, human-specific miR-21 mimics, miR-21 inhibitor, Rab27a and control siRNAs (GenePharma) were applied for the cultural system of SCP28 cells as previously described 35. MiR-21 mimics and inhibitors were synthesized by Gene Pharma (Shanghai, China). The sequences were listed as follow: human miR-21 mimics sequences: 5′-UAG CUU AUC AGA CUG AUG UUG A-3′; human miR-21 inhibitor sequences: 5′-UCA ACA UCA GUC UGA UAA GCU A-3′, control: CAG UAC UUU UGU GUA CAA; human Rab27a siRNA sequences: 5′-GCC AAU GGG ACA AAC AUA ATT-3′. To generate SCP28 cells with low expression of miR-21, the ShRNA with following sequence GAT CCG TAG CTT ACC AGG CTG ATG TTA ACT TCC TGT CAG ATC AAC ATC AGT CTG ATA AGC TAT TTT TG was used for miR-21 knockdown using a lentiviral transfection system as previously described 36.

### Co-culture assay

The well inserts with a 0.4-mm poresized filter (Corning Inc., USA) for six-well plates were used following the manufacturer's instruction. Approximately 4 × 10^6^ BMM cells were seeded into the bottom of each well and induced into osteoclasts in full α-MEM supplemented with 30 ng/mL M-CSF and 50 ng/mL RANKL for continuous 2 days. On the next day, 4 × 10^4^ SCP28 cells were seeded onto the top of transwell inserts and cultured separately in full DMEM overnight. Then, the induced BMM cells above were co-cultured together with SCP28 cells on the transwell inserts for another 2 days followed by TRAP staining (Sigma-Aldrich, USA) according to the manufacturer's instruction.

### Exosomes isolation and characterization

Exosomes were collected from cell culture supernatant by differential centrifugations as described previously 37. Briefly, SCP28 cells were cultured in exosomes-free DMEM medium described as above and the supernatant was collected at 48 to 72 h. Non-adherent cells were removed by centrifugation at 300 g for 10 min at 4 °C, and then the supernatant was collected in a new tube and performed secondary centrifugation at 2,000 g for 10 min at 4 °C. Next, the supernatant was transferred into a new tube followed by centrifugation at 10,000 g for 30 min at 4 °C. Finally, the supernatant was transferred into a new tube and pelleted exosomes via ultracentrifugation at 120,000 g for 70 min at 4 °C, followed by PBS washing and centrifuged at 120,000 g for another 70 min. The exosome pellet was resuspended in PBS at an appropriate volume. The concentration of exosome suspension was measured by a BCA protein assay kit (Beyotime, China). The characterization of exosomes was confirmed by measuring morphology with a HT7700 (Hitachi, Japan) transmission electron microscope (TEM) and particle size with LM10 NanoSight Tracking analysis (Malvern Instruments, Britain). Exosomes used for RNA and protein extraction were isolated using Total Exosome Isolation Kit (Invitrogen, USA).

### Analysis of exosome uptake by RANKL-primed osteoclast precursors

The fluorescent dye 3,3′-dioctadecy loxacar bocyanine perchlorate (Dio) (5 μM, Invitrogen, USA) was added to exosome suspension, which was incubated for 15 min at 37 °C. The pellet was washed twice with PBS followed by ultracentrifugation at 120,000 g for 70 min and then resuspended in PBS at an appropriate volume. RANKL-primed osteoclast precursors were incubated with the Dio-labeled exosomes for 4 h at 37 °C. Then, the cells were washed twice with PBS and fixed in 4% paraformaldehyde for 5 min at room temperature. Next, the cells were washed twice with PBS and incubated with Hoechst 33342 for 5 min at 37 °C. The labeled cells were examined using confocal laser scanning microscopy (LSM-710, Zeiss, Germany).

### *In vivo* and *in vitro* exosome treatment

*In vitro* osteoclast differentiation was accomplished as previously described 38. Briefly, BMM cells from femurs of 6 to 8 weeks old mice were cultured in full α-MEM with M-CSF (10 ng/mL, R&D systems, USA) for 1 d and then were differentiated into osteoclasts using RANKL (50 ng/mL, R&D systems, USA) and M-CSF (30 ng/mL) for 5 d. 50 μg/mL exosomes were added to 2 × 10^6^ recipient cells (TRAP^+^ monocytes) on the fourth day. For *in vivo* treatment, 50 μg exosomes were intravenously injected into 6-week-old female BALB/c nude mice every other day for 3 weeks. In the control group, PBS was used.

For the culture of osteoblast progenitor cells, calvariae from newborn mice were dissected aseptically and treated with 0.1% collagenase and 0.2% dispase. The cells were maintained in the minimum essential medium (MEM) alpha containing 10% FBS. For osteoblast differentiation, primary osteoblasts were cultured in α-MEM containing 10% FBS and 10 nM dexamethasone (Sigma), 50 µg/mL of ascorbic acid, and 5 mM β-glycerophosphate for 6 days. For *in vitro* treatment, 50 μg/mL of exosomes were added to 1 × 10^4^ recipient cells on the first day. Alkaline phosphatase (ALP) staining was carried out using a Vector Blue Substrate Kit (SK-5300; Vector Laboratories).

### Detect the distribution of exosomes in multiple organs

Exosome suspensions were incubated with the fluorescent dye 1,1'-Dioctadecyl-3,3,3',3'-tetramethylindocarbocyanine perchlorate (Dil) (10 μM, Beyotime, China) for 20 min at room temperature, subsequently washed twice with PBS via ultracentrifugation at 120,000 g for 70 min. Pelleted exosomes were resuspended in PBS. Dil-labeled exosomes were detected using a Maestro in-vivo imaging system (CRi, USA) in multiple organs, including heart, liver, spleen, lung, kidney, bone and brain. Exosomes pre-labeled using Dil (100 μg per mouse) were then intravenously injected into 6-week-old female BALB/c nude mice, which were fed with alfalfa-free diet for 1 week before the experiment. Thereafter, the mice were killed at 8 h after the injection and subjected to biophotonic imaging to determine the distribution of labeled exosomes.

### Pit formation assay

The pit formation assay was performed as previously described 39. Approximately 3×10^6^ BMM cells were seeded into each well of a Corning® Osteo Assay Surface 24-well Plate (3987, Corning Inc., USA) and cultured overnight in the presence of M-CSF (10 ng/mL). On the next day, 30 ng/mL M-CSF and 50 ng/mL RANKL were amended into culture medium of BMM cells to induce into osteoclasts for continuous 7 days, which were treated with PBS or exosomes on the fourth day. The culture medium was discarded on day 7 and the cell surface was washed with 10% bleach solution for 5 min at room temperature. The plate was then washed twice with ddH_2_O and left to dry at room temperature for 5 h. Finally, the resorbing area was imaged via a microscope and analyzed with Image J software.

### Western blot

Total protein of cells was extracted in lysis buffer (50 mM Tris, pH 7.5, 250 mM NaCl, 0.1% SDS, 2 mM dithiothreitol, 0.5% NP-40, 1 mM PMSF and protease inhibitor cocktail) at 4 °C for 30 min. Bone tissues were grinded with the mortar in liquid nitrogen and were lysed in lysis buffer at 4 °C for 30 min. Protein fractions were collected by centrifugation at 12,000 g, 4 °C for 10 min. Next, 10 µg protein was subjected to SDS-PAGE and transferred onto polyvinylidene fluoride membranes. The membranes were blocked with 5% BSA and incubated with specific antibodies overnight. A horseradish peroxidase-labeled secondary antibody was added and visualized using an enhanced chemiluminescence kit (Pierce, USA). The following antibodies were used: TFIIB (1:1000, CST, USA), LaminA/C (1:1000, CST, USA), β-actin (1:1000, CST, USA), Rab27a (1:1000, CST, USA), EphA2 (1:1000, CST, USA), HSP70 (1:1000, CST, USA), TSG101 (1:1000, Santa Cruz Biotechnology, USA), Alix (1:1000, CST, USA), NFATc1 (1:1000, CST, USA) and PDCD4 (1:1000, CST, USA) to examine the concentrations of proteins in the lysates, respectively. The ratios of the protein band intensities relative to that of β-Actin were calculated for each sample using Image J.

### RNA extraction and Real-time PCR

Total RNA from bone tissues or cells was extracted with TRIzol Reagent (Invitrogen, USA) and reverse transcribed with PrimeScript RT reagent Kit (TaKaRa, China) following the manufacturer's instructions. Real-time PCR was performed using SYBR Premix Ex Taq II Kit (Takara, China). *Gapdh* was used as a normalization control for mRNA and *U6* was used as a normalization control in miRNA measurements. All primers used are shown in **Table S1**.

### Small RNA sequencing and data analysis

Total RNA from breast cancer cells-secreted exosomes was extracted with Trizol regent using the RNeasy Micro Kit (QIAGEN, Germany) and quantified by a Qubit 3.0 Fluorometer (Life Technologies, USA). The high-quality RNA was used for the sequencing library construction. Small RNA libraries were constructed using NEBNext Multiplex Small RNA Library Prep Set for Illumina and the NEB standard protocol. The libraries were sequenced on an Illumina platform by Novogene corporation (Beijing, China).

For RNA-seq data analysis, the sequencing quality of raw data was assessed by FastQC. First, the low-quality reads and adaptors were removed by Trimmomatic. Next, clean data for each sample were aligned to the most recent miRBase database with remaining reads aligned to the most recent human genome. Highly expressed microRNA in breast cancer cells-secreted exosomes were obtained.

### Statistical analysis

All experiments are presented as the mean ± SEM. Student's *t*-test was used for statistical evaluations of two group comparisons. One-way analysis of variance (ANOVA) for more than two groups was performed for statistical analysis. Comparisons between Kaplan-Meier curves were performed using the log-rank test. Other comparisons were performed using two-way ANOVA. All statistical analyses were performed with Prism software (GraphPad prism for windows, version 6.0).

## Results

### Implantation of SCP28 breast cancer cells results in bone loss of recipient mice

**Figure 1:**
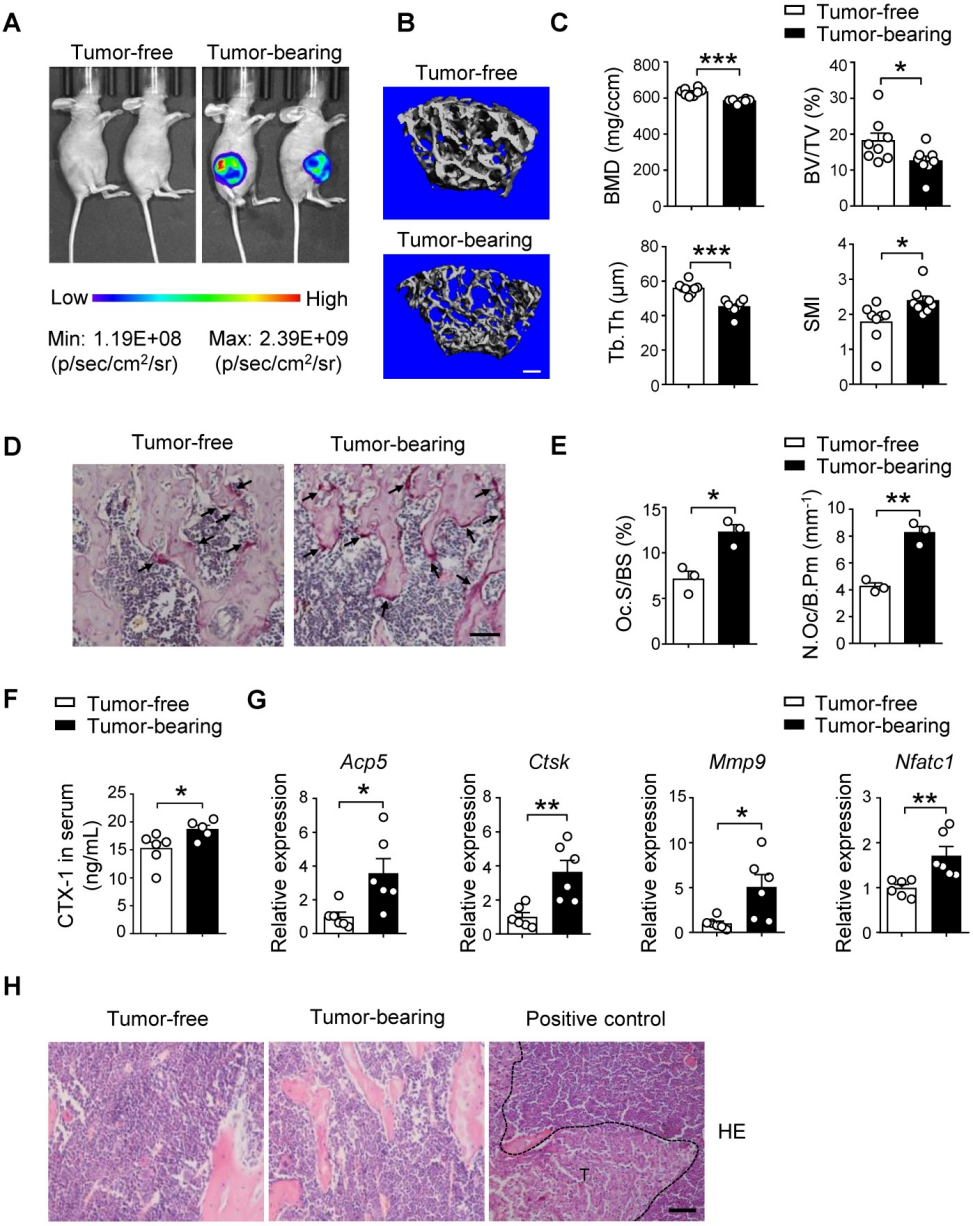
** Implantation of SCP28 breast cancer cells results in reduction of bone density. A.** Representative bioluminescent imaging (BLI) of recipients with SCP28 cells (right) or control mice (left) at checkpoint of 5 weeks post implantation. **B.** Representative images showing three-dimensional trabecular architecture by micro-computed tomography (micro-CT) reconstruction in the distal femurs of tumor-free and tumor-bearing mice (n = 8 mice per group). Scale bar, 300 µm. **C.** Quantification of BMD, BV/TV, Tb.Th, SMI from statistical micro-CT data. (n = 8 mice per group). BMD, bone mineral density; BV/TV, bone volume/tissue volume ratio; Tb.Th, trabecular thickness; SMI, structure model index. **D.** Representative images of TRAP-positive cells in trabecular bone of tibias from tumor-free and tumor-bearing mice. Scale bar, 50 µm. **E.** Osteoclast surface/bone surface (Oc.S/BS) and osteoclast number/bone perimeter (N.Oc/B.Pm) of the proximal tibia from distinct mice in D were indicated (n = 3 in each group). **F.** ELISA analysis of serum cross linked C-telopeptide of type 1 collagen (CTX-1) (ng/mL) in tumor-free (n = 6) and tumor-bearing mice (n = 5). **G.** qRT-PCR analysis of the expression of osteoclast marker genes including *Acp5*, *Ctsk*, *Mmp9,* and *Nfatc1* in tibias and femurs from tumor-free mice (n = 6) and tumor-bearing mice (n = 6). The relative expression of each target transcript (after normalization to the housekeeping *Gapdh* gene) in tumor-free mice was set as 1, and that in tumor-bearing mice was normalized, accordingly. **H.** Representative images of H&E staining of tibia section from tumor-free, tumor-bearing mice and mice with bone metastasis of SCP28 cells as a positive control at checkpoint of 5 weeks post implantation. T: tumor cell. Scale bar, 50 µm. Cumulative data are means ± SEM. * *P* < 0.05; ** *P* < 0.01; *** *P* < 0.001 (unpaired Student's *t* test). All data are from at least three independent experiments.

To elucidate the pre-metastatic niche formation for breast cancer bone-metastasis, we examined the changes of bone mass and structure after orthotopic implantation of luciferase and GFP-labeled SCP28 breast cancer cells, which is an MDA-MB-231 derived cell line with high bone metastatic ability 40. The distal femoral metaphysis from SCP28 tumor-bearing mice showed gradual decrease of trabecular bone mineral density (BMD) after 4 weeks of implantation (**Figure S1A**) and significant changes were detected from 5 weeks post implantation (**Figure 1A and Figure S1A**). Consistently, bones from tumor-bearing mice exhibited a remarkable reduction in trabecular bone volume (BV/TV) and trabecular thickness (Tb.Th), and increased structure model index (SMI) in comparison with the control mice (**Figure 1B-C**), but no difference in trabecular number (Tb.N) and trabecular separation (Tb.Sp) (**Figure S1B**). These results indicate that implantation of primary breast tumors leads to bone loss.

Given bone is a dynamic tissue that is maintained by a delicate balance between osteoclasts and osteoblasts. We next analyzed the osteoclast and osteoblast activities in recipients at 5 weeks post implantation. Tartrate resistant acid phosphatase (TRAP) staining analysis showed increased osteoclasts activities in tumor-bearing mice (**Figure 1D**). The results of histomorphometric analyses demonstrated increased Oc.S/BS and N.Oc/B.Pm (**Figure 1E**). The bone resorption marker cross linked C-telopeptide of type 1 collagen (CTX-1) in serum was also elevated (**Figure 1F**). Accordingly, the expression of osteoclast differentiation and function genes *Acp5, Ctsk*, *Mmp9*, and* Nfatc1* was much higher in the bone of tumor-bearing mice than those in the bone of tumor-free mice (**Figure 1G**). In contrast, there were no alterations of bone formation parameters, including Ob.S/BS and N.Ob/B.Pm, OCN activity and serum levels of the bone formation marker PINP in the two groups (**Figure S1C-E**). At this time point, tumor cells were not detectable in the bone and there were no changes in body weight of recipients implanted with SCP28 cells, indicating no bone-metastasis occurred (**Figure 1H and Figure S1F-G**). Collectively, these data suggest that the bone lesion takes place prior to bone metastasis of breast cancer cells due to implantation of SCP28 cells promoting the activities of osteoclasts.

### Breast cancer cell-derived exosomes promote osteoclast activity *in vivo*

**Figure 2:**
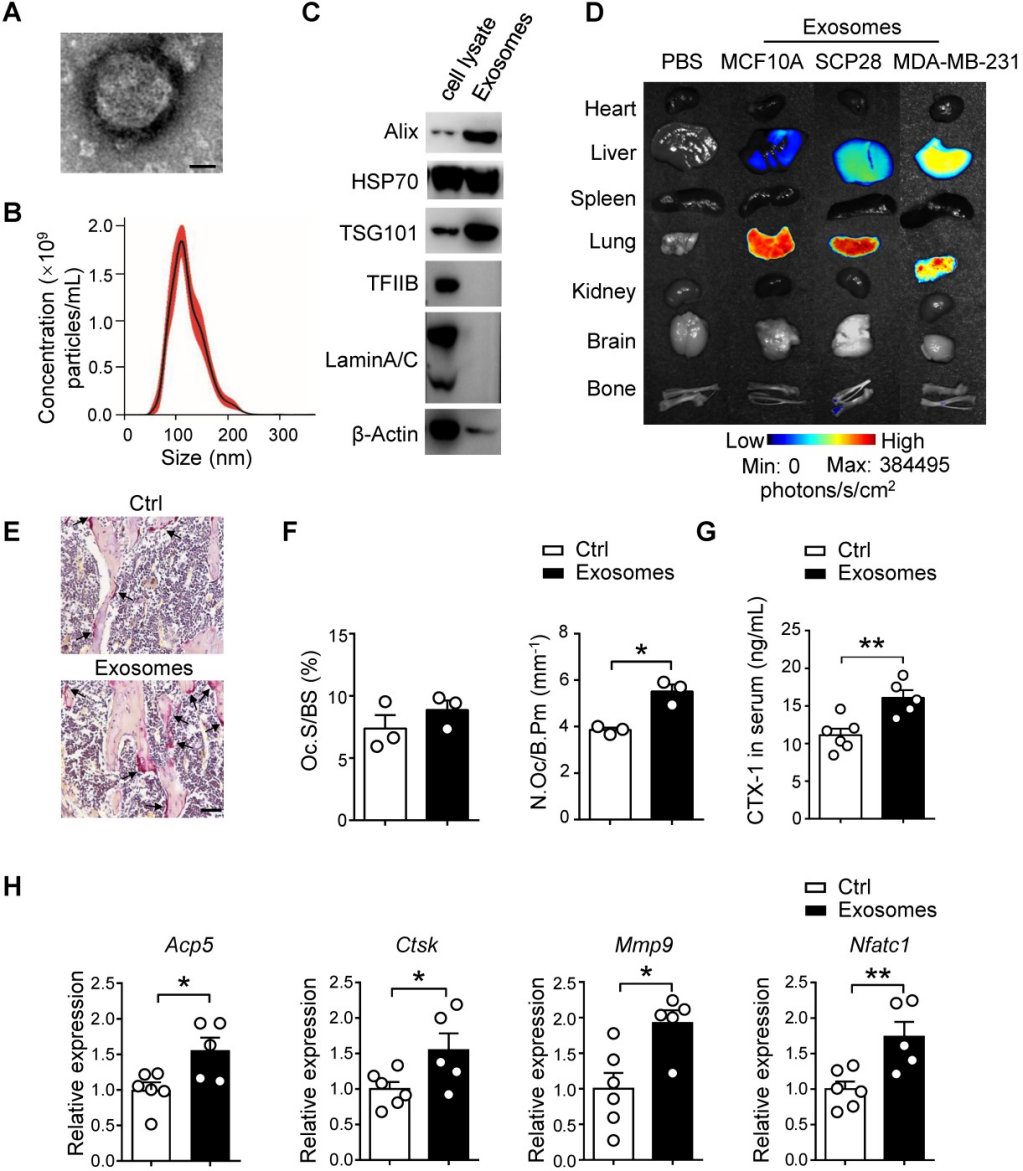
** SCP28 cell-secreted exosomes serve as a critical role in bone homeostasis. A.** Representative transmission electron microscopy (TEM) images of SCP28 cell-secreted exosomes. Scale bar, 25 nm. **B.** Nanoparticle tracking the size distribution of SCP28 cells-secreted exosomes (representative of five independent measurements). **C.** Detection of protein levels of Alix, HSP70, TSG101, TFIIB and LaminA/C in SCP28 cell-secreted exosomes and parental cell lysates by western blot. **D.** Representative BLI imaging showed the fluorescence signal distribution in multiple organs from mice injected with PBS or Dil-labeled exosomes derived from indicated cell lines. **E.** Representative images of TRAP staining in the proximal tibias of mice from indicated groups. Scale bar, 50 µm. **F.** Osteoclast surface/bone surface (Oc.S/BS) and osteoclast number/bone perimeter (N.Oc/B.Pm) of the proximal tibia from distinct mice in E were indicated (n = 3 in each group). **G.** ELISA analysis of serum CTX-1 (ng/mL) after administration with exosomes (n = 5) or PBS (n = 6), respectively. **H.** qRT-PCR analysis of osteoclast marker genes described as above in Oscar^+^ osteoclasts from mice treated with SCP28 cells-secreted exosomes (n = 5) and PBS as controls (n = 6). The relative expression of each target transcript (after normalization to the housekeeping *Gapdh* gene) in control mice was set as 1, and that in exosome-treated mice was normalized, accordingly. Cumulative data are means ± SEM. * *P* < 0.05; ** *P* < 0.01 (unpaired Student's *t* test). All data are from at least three independent experiments.

Owing to the critical functions of breast cancer cell-secreted exosomes as messengers between tumor cells and the bone niche of recipients, we isolated SCP28 cell-secreted exosomes to decipher their impact on bone microarchitecture. The exosomes were confirmed by transmission electron microscopy (TEM) (**Figure 2A**) and the purity of exosomes was measured by NanoSight analysis, revealing the range of exosome size was from 50 to 200 nm with an average exosome size of 110 ± 39 nm (**Figure 2B**). Exosome markers Alix, HSP70, and TSG101 were enriched, while nuclear proteins TFⅡB and Lamin A/C were undetectable in exosomes (**Figure 2C**). These data collectively verified that the purity of isolated exosomes was eligible for following experiments. We subsequently analyzed the distribution of exosomes derived from different cell lines including MCF10A, MDA-MB-231 or SCP28 cells in multiple organs of recipient mice at 8 h post intravenous injection. The result demonstrated that only SCP28 cell-secreted exosomes exhibited the propensity to reach the bone (**Figure 2D**), therefore we selected the SCP28 cell-secreted exosomes to perform the following assay.

To test whether exosomes derived from breast cancer cells have direct effects on osteoclast activity *in vivo*, SCP28 cell-secreted exosomes were intravenously injected into recipients every other day for 3 continuous weeks. There was no significant difference in body weight of all recipient mice after 3 weeks of exosome injection (**Figure S2A**). TRAP activity, serum CTX-1 level, and the expression of osteoclast marker genes *Acp5, Ctsk*, *Mmp9*, and* Nfatc1* in bones of exosome treated mice were up-regulated (**Figure 2E-G and Figure S2B**), reflecting osteoclast activity was substantially increased after exosome treatment. We further sorted the Oscar^+^ osteoclasts and detected the expression of osteoclast signature genes detected above. Consistently,* Acp5, Ctsk*, *Mmp9*, and* Nfatc1* were also up-regulated in exosome treated Oscar^+^ osteoclasts (**Figure 2H**). In contrast, there were no obvious effects on osteoblast number and activity from recipient mice treated with exosomes (**Figure S2C-E**).

### Breast cancer cell-derived exosomes promote osteoclast differentiation and activity* in vitro*

**Figure 3:**
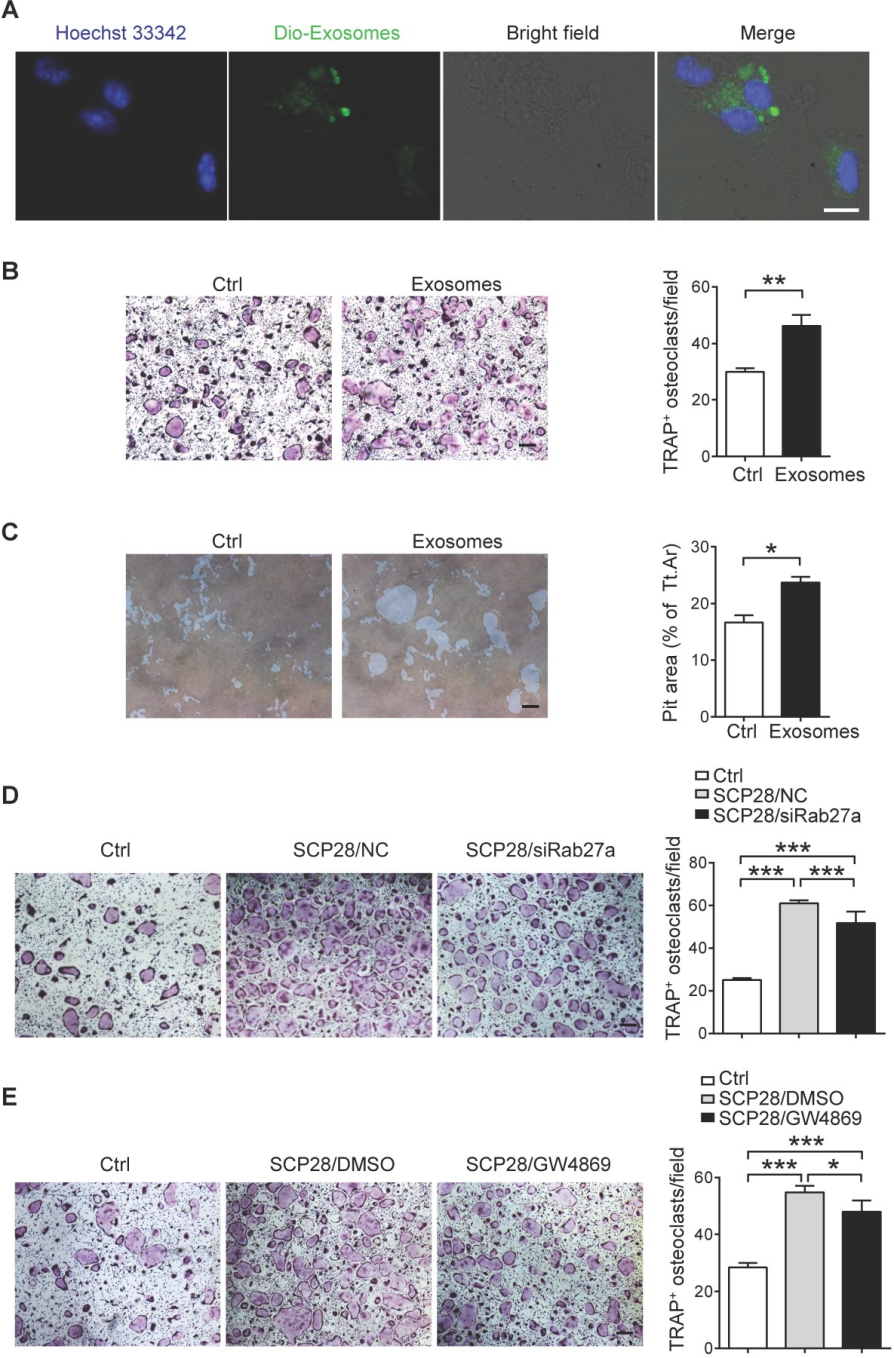
** SCP28 cell-secreted exosomes stimulate osteoclastogenesis *in vitro.* A.** Colocalization of SCP28 cell-secreted exosomes with RANKL-induced BMM cells using confocal microscopy imaging. Exosomes were labeled by 3'-dioctadecyloxacarbocyanine perchlorate (Dio, green) and cell nuclei were stained with Hoechst 33342 (blue). Scale bar, 10 µm. **B.** Representative TRAP staining images (left) and the number of osteoclasts with more than three nuclei (right) were statistically shown. Scale bar, 50 µm. ** *P* < 0.01 by Student's *t* test. **C.** Representative resorption pits (left) and cumulative data of pit resorption area (right) were statistically shown. Scale bar, 50 µm. Tt.Ar, total area. * *P* < 0.05 by Student's *t* test. **D.** Representative TRAP staining images (left) of RANKL-primed osteoclasts co-cultured with SCP28 cells treated by Rab27a siRNA or negative control (NC). Numbers of osteoclasts with more than three nuclei (right) were shown. Scale bar, 50 µm. NC, negative control. *** P < 0.001 by one-way ANOVA. **E.** Representative TRAP staining (left) of RANKL-primed osteoclasts co-cultured with SCP28 cells with or without N-SMase inhibitor GW4869 (20 µM). Numbers of osteoclasts with more than three nuclei (right) were shown. Scale bar, 50 µm. * *P* < 0.05; *** P < 0.001 by one-way ANOVA. Cumulative data are means ± SEM. The statistical methods are indicated relatively. All data are from at least three independent experiments.

We next analyzed the direct effects of breast cancer cells-derived exosomes on osteoclasts. Confocal imaging showed that Dio-exosomes could be incorporated into osteoclasts (**Figure 3A**). To further address the role of breast cancer-derived exosomes in osteoclast activation, primary osteoclast cells were cultured with or without the presence of SCP28 cell-secreted exosomes. The results showed that exosome treatment not only promoted the generation of TRAP-positive multinucleated osteoclasts (**Figure 3B**) and the expression of NFATc1 (**Figure S3A**), but also notably enhanced bone resorption *in vitro* (**Figure 3C**). We further confirmed the functions of SCP28 cell-secreted exosomes in osteoclast formation with Rab27a siRNA as an inhibitor of exosome secretion (**Figure S3B-C**) or the neutral sphingomyelinase inhibitor GW4869 via co-culture experiments, respectively. NanoSight analysis showed reduced amounts of exosomes secreted from SCP28 cells treated with Rab27a siRNA or GW4869 (**Figure S3D**). The number of TRAP-positive multinucleated osteoclasts was significantly reduced with treatment of Rab27a siRNA (**Figure 3D**) or GW4869 (**Figure 3E**), indicating that SCP28 cell-secreted exosomes could directly stimulate the formation of TRAP-positive multinucleated osteoclasts* in vitro.* In contrast, we observed exosomes treatment had no impact on the protein levels of osteoblast differentiation transactivator RUNX2 and ALP staining exhibited no significant difference (**Figure S3E-F**). Correspondingly, the mRNA levels of osteogenic genes *Alp*, *Ocn*, and *Col1* did not show statistical alteration in exosomes-treated osteoblasts compared with those from controls (**Figure S3G**). These data collectively demonstrate that SCP28 cell-secreted exosomes have a specific effect on osteoclast *in vitro*.

### Breast cancer cell-derived exosomes facilitate bone metastasis by priming pre-metastatic niche

**Figure 4:**
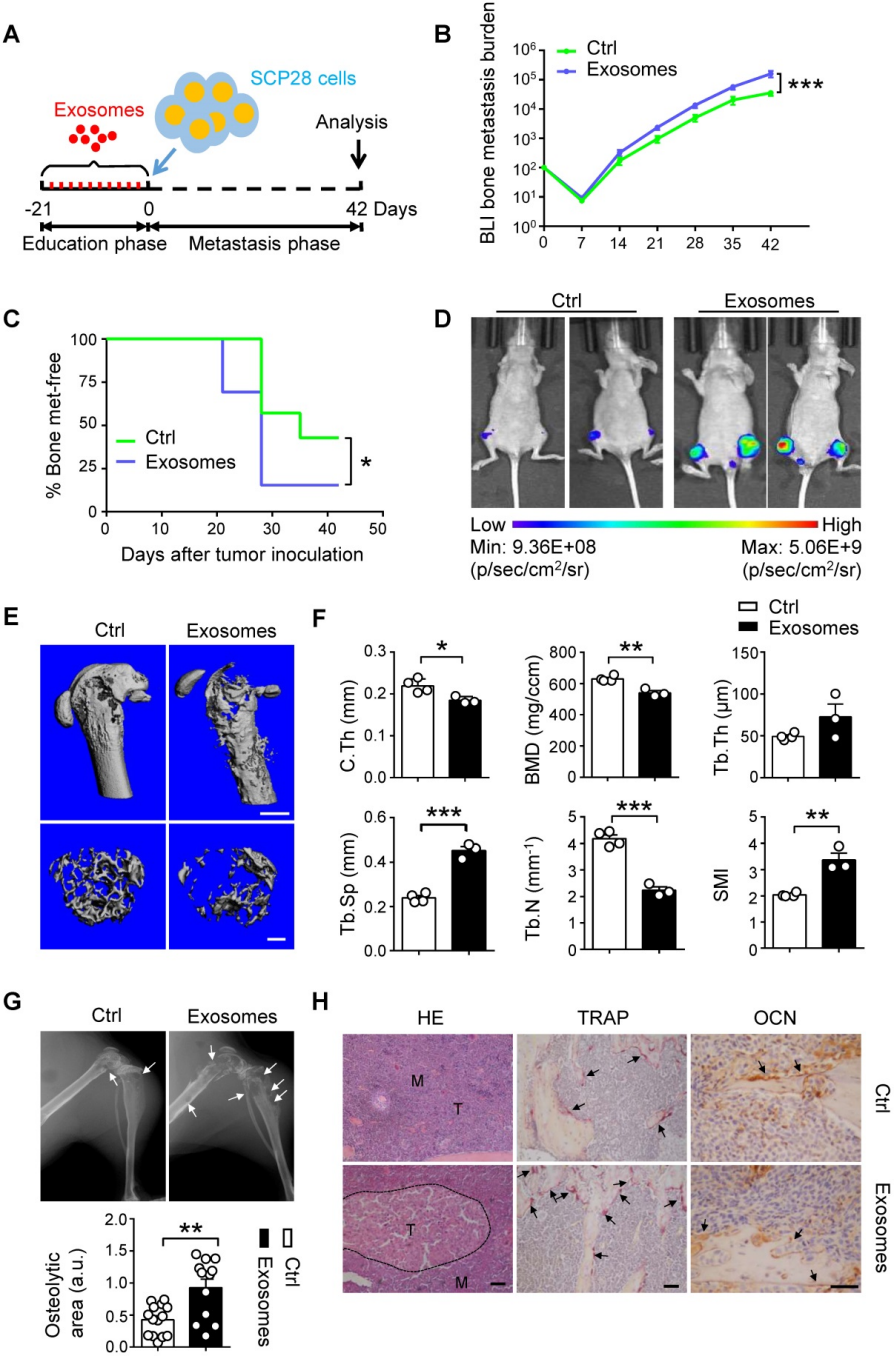
** SCP28 cells-secreted exosomes contribute to bone metastasis of SCP28 tumor cells. A.** Flowchart of the experimental processes and scheme of SCP28 cell-secreted exosome education and metastasis. **B.** BLI quantitation of dynamic bone metastasis of breast cancer SCP28 cells in recipient mice educated by SCP28 cell-secreted exosomes or controls (n = 14 per group); *** P < 0.001 by two-way ANOVA. **C.** Kaplan-Meier curve showing bone metastasis of SCP28 cells in recipient mice educated by SCP28 cell-secreted exosomes or controls (n = 14 per group); * P < 0.05 (log-rank test). **D.** Representative BLI imaging showing the SCP28 cells localization on day 42 in recipient mice educated by SCP28 cell-secreted exosomes or controls. **E.** Representative images showing three-dimensional architecture after micro-CT reconstruction of the distal femurs from mice. Scale bars, up 1 mm; bottom 300 µm. **F.** Quantitative micro-CT analysis of distal femurs from control (n = 4) and SCP28 exosomes mice (n = 3), including BMD, SMI, Tb.N, Tb.Th, Tb.Sp and C.Th. * *P* < 0.05; ** *P* < 0.01; *** *P* < 0.001 by Student's *t* test. **G.** Representative X-ray images (up) and quantification of osteolytic lesions (bottom) in SCP28 cell-implanted mice educated by SCP28 cell-secreted exosomes or controls. Arrows indicate osteolytic bone areas. ** P < 0.01 by Student's *t* test. a.u., arbitrary unit. **H.** Representative images of H&E, TRAP, and OCN staining from SCP28 cell-implanted mice educated by SCP28 cell-secreted exosomes or controls. T, tumor; M, bone marrow. Scale bars, 50 µm. Cumulative data are means ± SEM. The statistical methods are indicated relatively. All data are from at least three independent experiments.

To evaluate the effect of cancer cells-derived exosomes on the formation of pre-metastatic niche in the bone, we developed a well-established xenograft model by using SCP28 cells as donors for bone metastasis of recipients with or without treatment by SCP28 cell-secreted exosomes as described (**Figure 4A**). Tumor burden at the hind-limbs was significantly increased (**Figure 4B**) and the bone metastasis exhibited an earlier onset (**Figure 4C**) in the exosome-treated group. Notably, BLI imaging indicated that the tumor burden was significantly increased in recipients educated by exosomes compared with those in control group at the end point (**Figure 4D**). Reduced bone density and accelerated osteolysis of the cortical and trabecular bone were observed in recipients educated by exosomes (**Figure 4E-G**). Accordingly, immunohistochemistry staining reflected a significant increase of bone metastasis burden in exosomes-educated recipients (**Figure 4H**). Meanwhile, more TRAP positive osteoclasts at the tumor-bone interface were observed in recipients educated by exosomes, whereas the OCN staining indicated no changes in osteoblast numbers (**Figure 4H**).

To further validate these findings, we used mildly metastatic MDA-MB-231 cell line for implantation in mice after SCP28 cell-secreted exosomes education as described in scheme (**Figure S4A**). Substantially aggressive bone damage and elevated bone metastasis of MDA-MB-231 cells were observed at 6 weeks post tumor injection (**Figure S4B-H**). These data jointly indicate that SCP28 cell-secreted exosomes contribute to bone metastasis of breast cancer cells via accelerating osteoclast formation in bone microenvironment.

### Bisphosphonate treatment attenuates the osteoclast-driven pro-metastatic effect of breast cancer cell-derived exosomes in the bone

**Figure 5:**
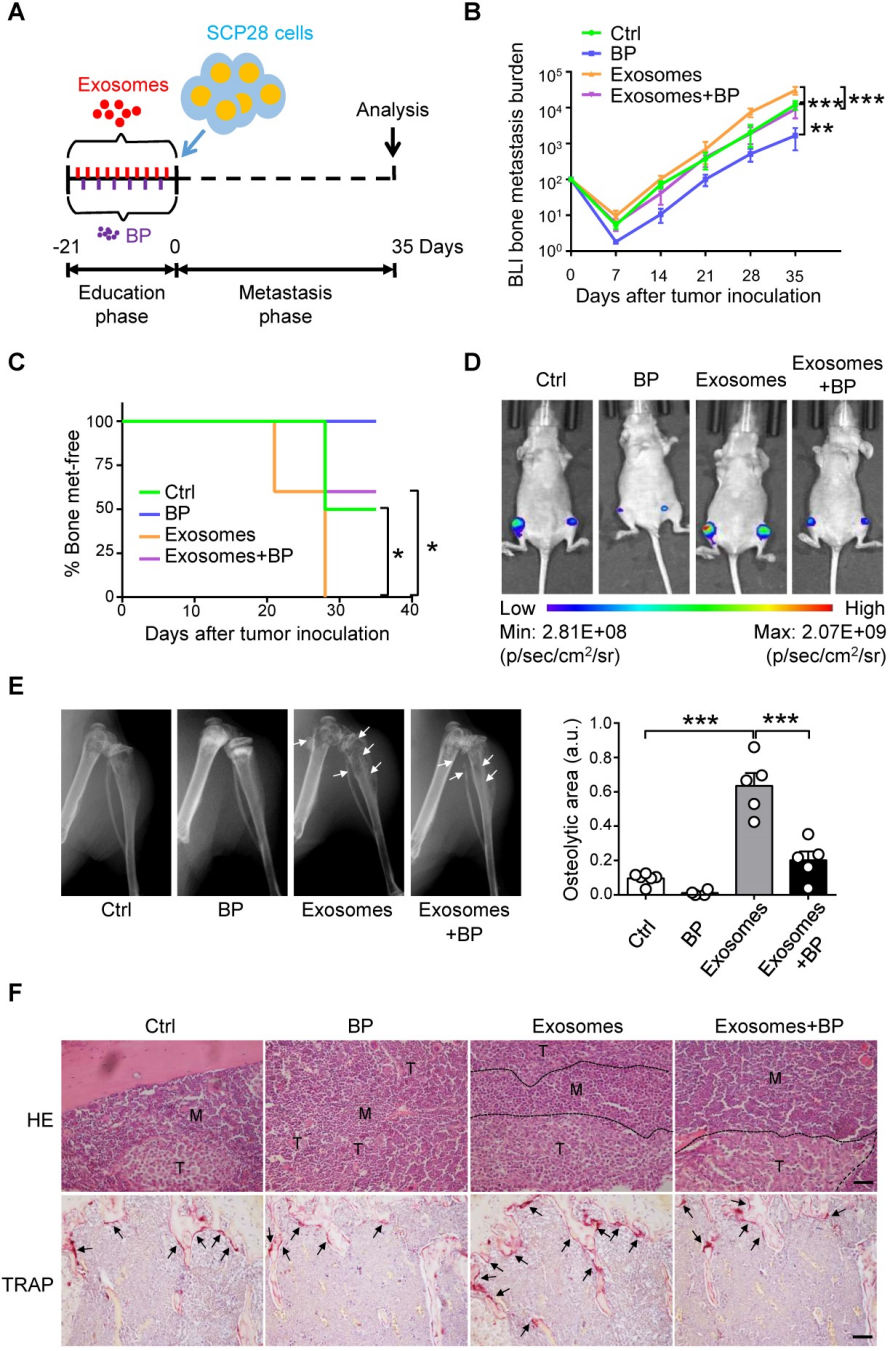
** SCP28 cells-secreted exosomes enhance bone metastasis by regulating osteoclast driven pre-metastatic niche. A.** Flowchart of the experimental processes and scheme of SCP28 cell-secreted exosome (treated with or without BP) education and analysis of metastasis. **B.** BLI quantitation of bone metastasis of SCP28 cells in recipient mice treated with exosomes or/and bisphosphonate zoledronic acid (BP) and controls. Control group, n = 6; BP group, n = 4; exosomes group, n = 6; exosomes + BP group, n = 5. ** *P* < 0.01, *** P < 0.001 by two-way ANOVA. **C.** Kaplan-Meier curve showing bone metastasis in recipient mice treated with control (n = 6), BP (n = 4), exosomes (n = 5), exosomes + BP (n = 5). * P < 0.05 (log-rank test). **D.** Representative BLI images showing the SCP28 cells localization on day 35 in recipient mice with different treatment. **E.** Representative X-ray images (left) and quantification of osteolytic lesions (right) in SCP28 cell-implanted mice with distinct treatment. Arrows indicate osteolytic bone areas. Control group, n = 6; BP group, n = 4; exosomes group, n = 5; exosomes + BP group, n = 5. *** P < 0.001 by one-way ANOVA. **F.** Representative images of H&E and TRAP staining of bones isolated from indicated mice on day 35 after exosomes or/and BP treatment. T, tumor; M, bone marrow. Scale bars, 50 µm. Cumulative data are means ± SEM. All data are from at least three independent experiments.

Next, we explored whether osteoclast is a direct downstream target of SCP28 cell-secreted exosomes in the establishment of pre-metastatic niche in bone. A series of experiments were developed by using exosomes-educated mice treated with or without bisphosphonate zoledronic acid (BP), an inhibitor of osteoclast function, following the experimental scheme (**Figure 5A**). Tumor burden at the hind-limbs was significantly decreased (**Figure 5B**) and the bone metastasis exhibited a late onset (**Figure 5C**) in recipients treated with exosomes and BP together than those in mice treated with exosomes only. Notably, BLI imaging indicated that the tumor burden was decreased in recipients treated with exosomes and BP compared with those in exosomes-educated mice (**Figure 5D**). Meanwhile, mice treated with exosomes and BP exhibited less osteolytic lesion formation (**Figure 5E and Figure S5A-B**). Accordingly, immunohistochemistry staining reflected a significant increase of bone metastasis burden in exosomes educated recipients, but a remarkable contraction in recipients treated with exosomes and BP (**Figure 5F**). TRAP positive osteoclast number at the tumor-bone interface exhibited the same trends, correspondingly (**Figure 5F**). Taken together, these data indicate that breast cancer cells-derived exosomes play essential roles in re-constructing the pre-metastatic niche via promoting osteoclast function.

### SCP28 cell-derived exosomal miR-21 promotes pre-metastatic niche formation via regulating osteoclastogenesis

**Figure 6:**
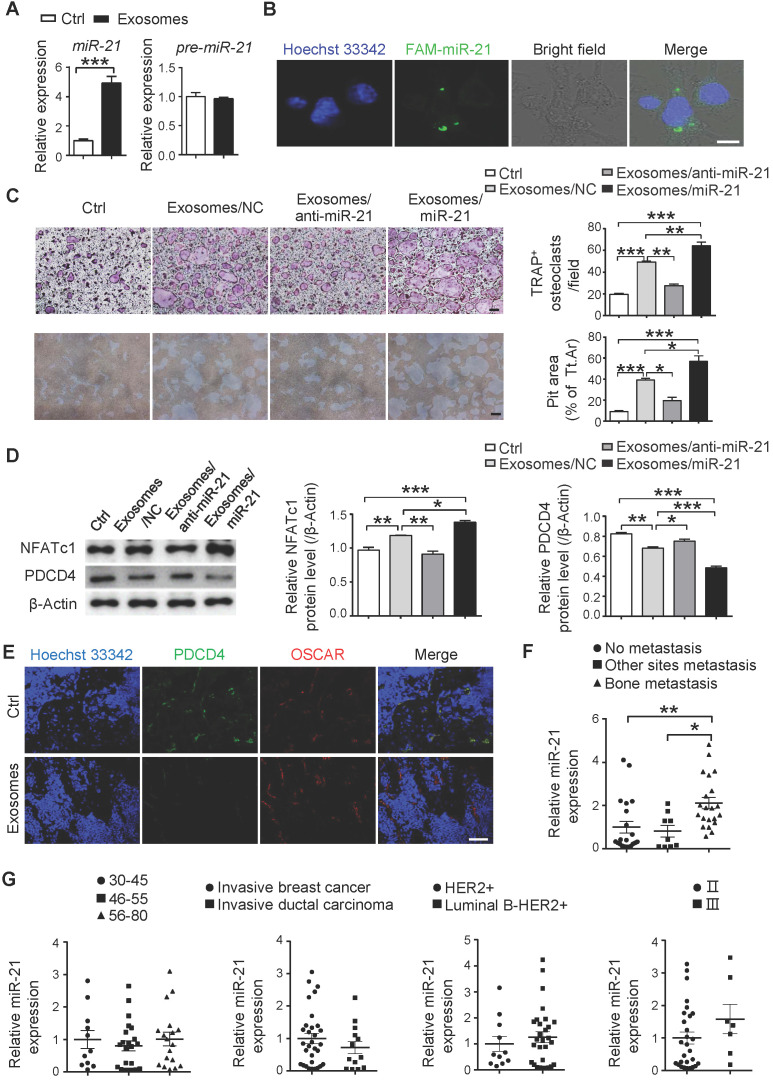
** SCP28 cell-derived exosomal miR-21 is involved in pre-metastatic niche formation via regulating osteoclast. A.** qRT-PCR analysis of expression of *miR-21* (miR-21-5p) and *pre-miR-21* levels in RANKL-primed osteoclasts treated with or without SCP28 cell-secreted exosomes for 6 h. The relative expression of *miR-21* or *pre-miR-21* was normalized to *U6* or *Gapdh* gene which is set as 1, respectively. *** P < 0.001 by Student's *t* test. **B.** Confocal microscopy images showing the transition of FAM-miR-21 from exosomes into osteoclasts at 6 h post treatment. miR-21 in exosomes from SCP28 cells were labeled by FAM (green) and cell nuclei were stained with Hoechst 33342 (blue). Scale bar, 10 µm. **C.** Representative TRAP staining images and resorption pits (left). The number of osteoclasts with more than three nuclei and cumulative data of pit resorption area (right) were statistically shown. Scale bars, 50 µm. ** *P* < 0.01, *** P < 0.001 by one-way ANOVA. **D.** Western blot (left) and quantitative analysis (right) of PDCD4 and NFATc1 protein expression in osteoclasts with indicated treatment. **E.** Confocal microscopy images showing the expression levels of PDCD4 and OSCAR in tibias section from recipient mice with indicated treatment. Scale bar, 10 µm. **F.** qRT-PCR analysis showing the expression level of *miR-21* in serum exosomes of breast cancer patients. No-metastasis patients, n = 21; other sites metastasis patients, n = 9; bone-metastasis patients, n = 21. The relative expression of *miR-21* was normalized to *cel-miR-39*. * P < 0.05, ** P < 0.01 by one-way ANOVA. **G.** Analysis of the level of *miR-21* in serum exosomes of breast cancer patients. 30-45, n = 11; 46-55, n = 22; 56-80, n = 18. Invasive breast cancer, n = 31; invasive ductal carcinoma, n = 13. HER2+, n = 11; Luminal B-HER2+, n = 29. II, n = 28; III, n = 7. The relative expression of *miR-21* was normalized to *cel-miR-39*. Cumulative data are means ± SEM. All data are from at least three independent experiments.

To explore the mechanisms of the SCP28 cell-secreted exosomes in regulating osteoclastogenesis and function, we analyzed the miRNA profiles of exosomes derived from SCP28 cancer cells. The miRNA sequencing results showed that many miRNAs were encapsulated in the exosomes (**Figure S6A and Table S2**). We selected the top 18 miRNAs enriched in the exosomes and analyzed their levels in RANKL-primed osteoclasts co-cultured with or without exosomes. Among these 18 miRNAs, miR-21 (miR-21-5p) exhibited the most significant changes with four-fold upregulation compared with control (**Figure 6A and Figure S6B**), while the level of pre-miR-21 was not altered (**Figure 6A**), suggesting that the increased miR-21 in osteoclasts was transported from exosomes, but not an intrinsically inducible expression. In addition, the expression of exosomal miR-21 in SCP28 cells was significantly higher than that in MCF10A, MDA-MB-231-LM2, and MDA-MB-231 cells (**Figure S6C**), implying miR-21 expression level was likely to be associated with the high metastatic capability of SCP28 cells. We further verified the transportation of miR-21 from exosomes to RANKL-primed osteoclasts via labeling miR-21 with FAM in exosomes (FAM-miR-21). Confocal imaging showed the presence of FAM-miR-21 in the cytoplasm of osteoclasts, indicating that SCP28 cell-derived miR-21 could be delivered into osteoclasts from exosomes (**Figure 6B**).

To further confirm the effect of exosomal miR-21 on osteoclast, exosomes were isolated from SCP28 cells transfected with miR-21 siRNA or mimics, and miR-21 levels were confirmed (**Figure S6D**). When RANKL-primed osteoclasts were incubated with exosomes secreted by SCP28 cells transfected with miR-21 siRNA (exosomes/anti-miR-21), TRAP positive multi-nuclear osteoclast numbers were reduced and the resorption capability of osteoclast was compromised in comparison with those exosomes from the control group (**Figure 6C**). Conversely, the effect of exosomes on osteoclasts was reinforced by exosomes purified from SCP28 cells transfected with miR-21 mimics (exosomes/miR-21) (**Figure 6C**).

Given the PDCD4, a direct target of miR-21, serves as an inhibitor in osteoclast differentiation and function, we next confirmed the expression of PDCD4 and NFATc1 from different experimental groups. As predicted, the decreased NFATc1 expression and increased PDCD4 expression were observed in osteoclasts treated with exosomes/anti-miR-21 and the opposite effects were exhibited in those treated with exosomes/miR-21 (**Figure 6D**). Consistently, immunohistochemistry staining of the bone from exosome-educated mice revealed a significantly lower PDCD4 protein levels in OSCAR^+^ osteoclasts in comparison with the control group (**Figure 6E**).

To evaluate the effect of cancer cells-derived exosomal miR-21 on the formation of a pre-metastatic niche *in vivo*, SCP28 cells transfected with Sh-miR-21 or NC were injected into mammary fat pads of mice to establish the primary tumors-bearing mice models. The knockdown efficiency of Sh-miR-21 was confirmed by qRT-PCR (**Figure S6E**). The bone density of the femur from SCP28 tumor-bearing mice was further analyzed by micro-CT at 5 weeks post transplantation. The degree of bone loss in recipient mice bearing SCP28/Sh-miR-21 tumors was significantly lower than that in recipient mice bearing SCP28/NC tumors (**Figure S6F-G**). Correspondingly, serum CTX-1 level and the expression of osteoclast signature genes *Acp5, Ctsk*, *Mmp9*, and* Nfatc1* in mice bearing SCP28/Sh-miR-21 tumors were comparable with tumor-free mice (**Figure S6H-I**). These data suggested miR-21 played a crucial role in osteoclast-driven pre-metastatic niche formation. Importantly, exosomal miR-21 levels in the serum from breast cancer patients with bone-metastasis were dramatically higher than that from breast cancer patients with non-bone tissue metastasis and without metastasis, while exosomal miR-21 levels in the serum had no significant correlation with age, stage and subtypes of breast cancer patients (**Figure 6F-G and Table S3**).

## Discussion

The cross-talk between tumor cells and the bone microenvironment linking to bone metastasis of breast cancer has captivated widely research interests in the past few years. Recent studies indicate that exosomes-mediated exchange of cellular materials between cells functions as an important intercellular communication 41-43. It has been intensively described that tumor cell-derived exosomes play critical roles in tumor progression and aggressiveness, particularly inducing pre-metastatic niche formation and consequently increasing metastatic burden 44. Exosomes derived from metastatic tumor help to establish a favorable local microenvironment in lung that supports survival and outgrowth of disseminated breast cancer 45, 46. Similarly, breast cancer cells-secreted exosomes also contribute to pre-metastatic niche formation in the brain by recruiting immune cells and influencing metabolic reprogramming of stromal cells 18. Here, we demonstrated that breast cancer cells-secreted exosomes contribute to bone metastasis by the establishment of the pre-metastasis microenvironment in bone tissues.

Despite accumulating evidence has shown that extracellular vesicles are essential to the metastatic process for the delivery of active molecules to bone 39, 47, 48, the underlying mechanisms in bone metastases of breast cancer are still unclear. We found that SCP28 cells, a high metastatic cell line, secreted exosomes exhibiting a higher migration capacity into bone tissue compared with exosomes derived from MDA-MB-231 cells. Meanwhile, mice treated with SCP28 cell-secreted exosomes displayed a severe bone loss, which was tightly associated with bone metastatic burden. Accordingly, bisphosphonate treatment attenuated the pro-metastatic effect of breast cancer cells-derived exosomes in the bone, suggesting breast cancer-secreted exosomes have a central role in the establishment of pre-metastatic niche for bone metastasis of breast cancer cells.

Pre-metastatic niche formation is correlated with aberrant bone remodeling, which is a precise physiological process requiring the balance of osteoblasts and osteoclasts. Emerging studies have demonstrated that exosomes secreted by cancer cells have the capacities in regulating osteoclast activity, including multiple myeloma, acute myeloid leukemia and lung cancer 49-51. To address the mechanism of bone remodeling caused by breast cancer cells-derived exosomes, we performed a series of experiments to identify that exosomes can transfer into osteoclasts to stimulate their differentiation and activity. Our results from both* in vitro* and *in vivo* assays revealed that breast cancer‐derived exosomes promoted osteoclast formation to establish the pre-metastatic microenvironment, but had no effect on osteoblasts. The active material with physiological function in exosomes is dominantly composed of nuclear acid and protein 52. Tiedemann and colleagues reported that exosomal release of L-Plastin by MDA-MB-231 cells influences osteoclasts 53. In addition, primary breast tumor-secreted hypoxic secretome lysyl oxidases acts directly on osteoclasts in the absence of bone metastasis, leading to focal osteolytic lesions formation in 4T1 tumor cells-bearing mice 54. Collectively, distinct contents derived from different tumor cells serve as messengers via directly targeting osteoclasts for establishment of metastatic niche.

To achieve the goal of deciphering the regulators from breast cancer‐derived exosomes, we performed the miRNA seq analysis in SCP28 cell-secreted exosomes and relative validation in exosomes-treated osteoclasts. We found that miR-21 was expressed at an absolutely high level. As a potential target, we experimentally verified that exosomal miR-21 could be internalized by osteoclasts and promoted their differentiation and function. As a key regulator of osteoclast, the expression level of PDCD4 was significant lower in osteoclasts treated with exosomes derived from breast cancer cell, indicating exosomal miR-21 was likely to regulate the establishment of metastatic niche via directly targeting PDCD4.

Given miR-21 is significantly higher in the plasma exosomes of patients with breast cancer compared with healthy control subjects 55, we then measured the expression level of exosomal miR-21 in serum exosomes from breast cancer patients. Our results demonstrated that breast cancer patients with bone metastasis showed higher expression of miR-21 in serum exosomes than those without metastasis or with non-bone tissue metastasis, providing essential implications for the efficient non-invasive diagnosis. Furthermore, miR-21 level in serum exosomes was irrelevant to age, grade and subtype of breast cancer patients based on our patient samples.

In summary, we demonstrate that breast cancer-secreted exosomes function as critical media for establishing a pre-metastatic niche to accelerate bone metastasis of tumor cells. During this process, exosomal miR-21 derived from cancer cells serves as an intercellular messenger for osteoclast differentiation and function by regulating PDCD4 expression. Furthermore, the relative correlation in patient samples strengthens the clinical significance of miR-21 as a potential target linking to breast cancer bone-metastasis. Thus, our study not only demonstrates an underlying mechanism of breast cancer bone metastasis, but also gives rise to a clinical insight for therapeutic inhibition of miR-21 in osteoclasts towards translational medicine.

## Supplementary Material

Supplementary figures and table S1.

Supplementary table S2.

Supplementary table S3.

## Abbreviations

PDCD4: programmed cell death 4
H&E: hematoxylin-eosin
TRAP: tartrate resistant acid phosphatase
BLI: bioluminescent imaging
Micro-CT: Micro-computed tomography
OCN: osteocalcin
Oc.S/BS: osteoclast surface/bone surface
N.Oc/B.Pm: osteoclast number/bone perimeter
Ob.S/BS: osteoblast surface/bone surface
N.Ob/B.Pm: osteoblast number/bone perimeter
BMD: bone mineral density
BV/TV: bone volume per tissue volume
Tb.Th: trabecular thickness
SMI: structure model index
Tb.N: trabecular number
Tb.Sp: trabecular separation
C.Th: cortical thickness
CTX-1: cross linked C-telopeptide of type 1 collagen
PINP: I type collagen amino end before collagen peptide
TEM: transmission electron microscopy
BMM: bone marrow monocyte
Dio: 3,3'-dioctadecyloxacarbocyanine perchlorates
MEM: minimum essential medium
ALP: Alkaline phosphatase
Dil: 1,1'-Dioctadecyl-3,3,3',3'-tetramethylindocarbocyanine perchlorate
Tt.Ar: total area
BP: bisphosphonate zoledronic acid
