# Supplementary material for: Breast cancer exosomes contribute to pre-metastatic niche formation and promote bone metastasis of tumor cells
Source: Theranostics. 2021 Jan 1;11(3):1429–45. doi: 10.7150/thno.45351 (PMC7738874; doi:10.7150/thno.45351)
Supplement: Supplementary file 1 — Supplementary figures and table S1. [file thnov11p1429s1.pdf]

**Supplemental Information for:**

# Breast cancer exosomes contribute to pre-metastatic niche formation and promote bone metastasis of tumor cells

Xinxin Yuan[1, 2 #], Niansong Qian[3, #], Shukuan Ling[2], Yuheng Li[2], Weijia Sun[2], Jianwei Li[2], Ruikai Du[2], Guohui Zhong[2], Caizhi Liu[2], Guotao Yu[1], Dengchao Cao[1, 2], Zizhong Liu[2], Yinbo Wang[2], Zhihong Qi[1], Yingpeng Yao[1], Fang Wang[1], Jingjing Liu[1], Shanshan Hao[1], Xiaoyan Jin[2], Yinlong Zhao[2], Jianqi Xue[2], Dingsheng Zhao[2], Xingcheng Gao[2], Shuai Liang[2], Youyou Li[2], Jinping Song[2], Shuyang Yu[1, *], and Yingxian Li[2, *]

1 State Key Laboratory of Agrobiotechnology, College of Biological Sciences, China Agricultural University, Beijing 100193, China

2 State Key Lab of Space Medicine Fundamentals and Application, China Astronaut Research and Training Center, Beijing 100094, China

3 Department of Oncology, Chinese PLA General Hospital, Beijing 100853, China

# Equal contributions to this work

* Corresponding authors:

Prof. Yingxian Li, Email: yingxianli@aliyun.com

Prof. Shuyang Yu, Email: ysy@cau.edu.cn

**Supplementary Figure 1. The influence of primary tumor on osteoblasts before the occurrence of bone metastasis**

**A.** Quantitative micro-CT analysis of BMD of recipient mice at different checkpoint from the indicated groups (n = 6 mice per group).

**B.** The values of micro-CT parameters (Tb.N, Tb.Sp) at the distal femur metaphysis from tumor-free and tumor-bearing mice. (n = 8 mice per group). Tb. N, trabecular number, Tb.Sp, trabecular separation.

**C.** Osteoblast surface/bone surface (Ob.S/BS) and osteoblast number/bone perimeter (N.Ob/B.Pm) of the proximal tibia of recipient mice from the indicated groups (n = 3 in each group).

**D.** Representative images of the osteocalcin (OCN) staining in tibia sections from tumor-free and tumor-bearing mice. Scale bar, 25 μm.

**E.** ELISA analysis of the bone formation marker PINP levels in serum of tumor-free (n = 6) and tumor-bearing mice (n = 5).

**F.** Representative images of anti-GFP staining of tibia section from tumor-free, tumor-bearing mice and mice with bone metastasis of SCP28 cells as a positive control at checkpoint of 5 weeks post implantation. The brown areas represent tumor. Scale bar, 50 μm.

**G.** The changes of body weight in recipient mice at different checkpoint from tumor-free (n = 8) and tumor-bearing mice (n = 7).

Cumulative data are means ± SEM. * $P < 0.05$; ** $P < 0.01$; *** $P < 0.001$ (unpaired Student's *t* test). All data are from at least three independent experiments.

**Supplementary Figure 2. The effect of SCP28 cell-secreted exosomes on osteoclasts and osteoblasts *in vivo***

**A.** Body weight of recipient mice treated with SCP28 exosomes or PBS (n = 6 in each group).

**B.** Analysis of the expression of osteoclast marker genes including *Acp5*, *Ctsk*, *Mmp9,* and *Nfatc1* in tibias and femurs from mice treated with exosomes (n = 5) or PBS as controls (n = 6), respectively. The relative expression of each target transcript (after normalization to the housekeeping *Gapdh* gene) in control mice was set as 1, and that in exosome-treated mice was normalized, accordingly.

**C.** Ob.S/BS and N.Ob/B.Pm of the proximal tibia from the indicated groups (n = 3 in each group).

**D.** Representative images of the OCN staining in tibia sections from recipient mice treated with SCP28 exosomes or PBS. Scale bar, 25 μm.

**E.** ELISA analysis of the level of PINP in serum of recipient mice treated with SCP28 exosomes (n = 5) or PBS (n = 6).

Cumulative data are means ± SEM. * $P < 0.05$ (unpaired Student's *t* test). All data are from at least three independent experiments.

**Supplementary Figure 3. The effect of SCP28 cell-secreted exosomes on osteoclasts and osteoblasts *in vitro***

**A.** Western blot analysis of expression of NFATc1 in BMM cell induced osteoclasts treated with or without SCP28 cell-secreted exosomes.

**B.** *Rab27a* mRNA level was analyzed in SCP28 cells treated with Rab27a siRNA or NC. The relative expression of *Rab27a* (after normalization to the housekeeping *Gapdh* gene) in NC samples was set as 1, and that in *siRab27a* samples was normalized, accordingly. *** $P < 0.001$ by Student's *t* test.

**C.** Western blot analysis of expression of NFATc1 in SCP28 cells treated with *Rab27a* siRNA or NC.

**D.** The amounts of exosomes secreted by SCP28 cells treated with *Rab27a* siRNA or GW4869 was analyzed by NanoSight.

**E.** Western blot analysis of expression of RUNX2 in osteoblasts treated with or without SCP28 cell-secreted exosomes.

**F.** Representative images of ALP staining in osteoblasts treat with SCP28 exosomes or not. Scale bar, 3 mm.

**G.** qRT-PCR analysis of *Alp*, *Col1α1,* and *Ocn* mRNA in osteoblasts treated with or without SCP28 cell-secreted exosomes. The relative expression was normalized to *Gapdh* gene which is set as 1. Cumulative data are means ± SEM. *** $P < 0.001$ (unpaired Student's *t* test). All data are from at least three independent experiments.

**Supplementary Figure 4. SCP28 cells-secreted exosomes enhance bone metastasis of MDA-MB-231 tumor cells**

**A.** Flowchart of the experimental processes and scheme of SCP28 cell-secreted exosome education and metastasis

**B.** BLI quantitation of dynamic bone metastasis of breast cancer MDA-MB-231 cells in recipient mice educated by SCP28 cell-secreted exosomes (n = 6) or controls (n = 7); ** $P < 0.01$ by two-way ANOVA.

**C.** Kaplan-Meier curve showing bone metastasis of MDA-MB-231 cells in recipient mice educated by SCP28 cell-secreted exosomes (n = 6) or controls (n = 7); * $P < 0.05$ (log-rank test).

**D.** Representative BLI imaging showing the MDA-MB-231 cells localization on day 42 in recipient mice educated by SCP28 cell-secreted exosomes or controls.

**E.** Representative images showing three-dimensional architecture after micro-CT reconstruction of the distal femurs from recipient mice. Scale bars, up 1 mm; bottom 300 μm.

**F**. Quantitative micro-CT analysis of distal femurs from recipient mice educated by SCP28 cell-secreted exosomes (n = 5) or controls (n = 7), including BMD, SMI, Tb.N, Tb.Th, Tb.Sp and C.Th. * $P < 0.05$; ** $P < 0.01$ by Student's *t* test.

**G.** Representative X-ray images (up) and quantification of osteolytic lesions (bottom) in MDA-MB-231 cell-implanted mice educated by SCP28 cell-secreted exosomes or controls (n = 7 per group). Arrows indicate osteolytic bone areas. ** $P < 0.01$ by Student's *t* test.

**H.** Representative images of H&E, TRAP, and OCN staining from MDA-MB-231 cell-implanted mice educated by SCP28 cell-secreted exosomes or controls. T, tumor; M, bone marrow. Scale bar, 25 μm.

Cumulative data are means ± SEM. The statistical method indicated relatively. All data are from at least three independent experiments.

**Supplementary Figure 5. Bisphosphonate treatment attenuates pro-metastatic effect of breast cancer cell-derived exosomes in the bone**

**A.** Representative images showing three-dimensional architecture after micro-CT reconstruction of the distal femurs from recipient mice. Scale bars, up 1 mm; bottom 300 μm.

**B**. Quantitative micro-CT analysis of distal femurs from control recipient mice with different treatment: Control, n = 5; BP, n = 4; exosomes, n = 5; exosomes + BP, n = 5, including BMD, SMI, Tb.N, Tb.Th, Tb.Sp and C.Th. * $P < 0.05$; ** $P < 0.01$; *** $P < 0.001$ by one-way ANOVA.

Cumulative data are means ± SEM. All data are from at least three independent experiments.

**Supplementary Figure 6. SCP28 cell-derived exosomal miR-21 promotes osteoclastogenesis**

**A.** Heatmap globally showing enriched miRNAs in SCP28 cell-secreted exosomes. Normalized expression level of miRNAs which are higher in the top 200 miRNAs was labeled on the top and the scale bar on the right.

**B.** Normalized miRNA expression levels for the top 17 miRNAs except *miR-21-5p* in SCP28 cell-secreted exosomes. The relative expression was normalized to *U6* gene which is set as 1. * $P < 0.05$; ** $P < 0.01$; ***, $P < 0.001$ by Student's *t* test.

**C.** qRT-PCR analysis of *miR-21* level in exosomes secreted by MCF10A, MDA-MB-231, SCP28 and parental MDA-MB-231-LM2 cells. The relative expression was normalized to *miR-16* gene which are set as 1, respectively. * $P < 0.05$; ** $P < 0.01$ by one-way ANOVA.

**D.** The expression of *miR-21* in SCP28 cells and exosomes was measured by qRT-PCR. The relative expression of *miR-21* in cell or exosomes was normalized to *U6* or *miR-16* gene which are set as 1, respectively. * $P < 0.05$; ** $P < 0.01$ by one-way ANOVA

**E.** qRT-PCR analysis of *miR-21* level in SCP28 cells treated with miR-21 ShRNA or NC. The relative expression of *miR-21* was normalized to *U6* gene which is set as 1. ** $P < 0.01$ by Student's *t* test.

**F.** Representative images showing three-dimensional trabecular architecture after micro-CT reconstruction of the distal femoral metaphysis of mice. Scale bar, 300 μm.

**G.** Quantitative micro-CT analysis of distal femurs from mice in each experimental group, including BMD, SMI, Tb.Th, Tb.Sp and BV/TV. Control, n = 7; SCP28/NC, n = 6; SCP28/Sh-miR-21, n = 6. * $P < 0.05$; ** $P < 0.01$; *** $P < 0.001$ by one-way ANOVA.

**H.** ELISA analysis of serum CTX-1 (ng/mL) in control (n = 7), SCP28/NC (n = 6) and SCP28/Sh-miR-21 (n = 7) mice. ** $P < 0.01$ by one-way ANOVA.

**I.** qRT-PCR analysis of the expression of osteoclast marker genes including *Acp5*, *Ctsk*, *Mmp9,* and *Nfatc1* in tibias and femurs from control (n = 6), SCP28/NC (n = 5) and SCP28/Sh-miR-21 (n = 5) mice. The relative expression of each target transcript (after normalization to the housekeeping *Gapdh* gene) in tumor-free mice was set as 1, and that in tumor-bearing mice was normalized, accordingly. *

*P* < 0.05; ** *P* < 0.01; *** *P* < 0.001 by one-way ANOVA.

Cumulative data are means ± SEM. All data are from at least three independent experiments.

# Yuan et al Supplementary Figure 1

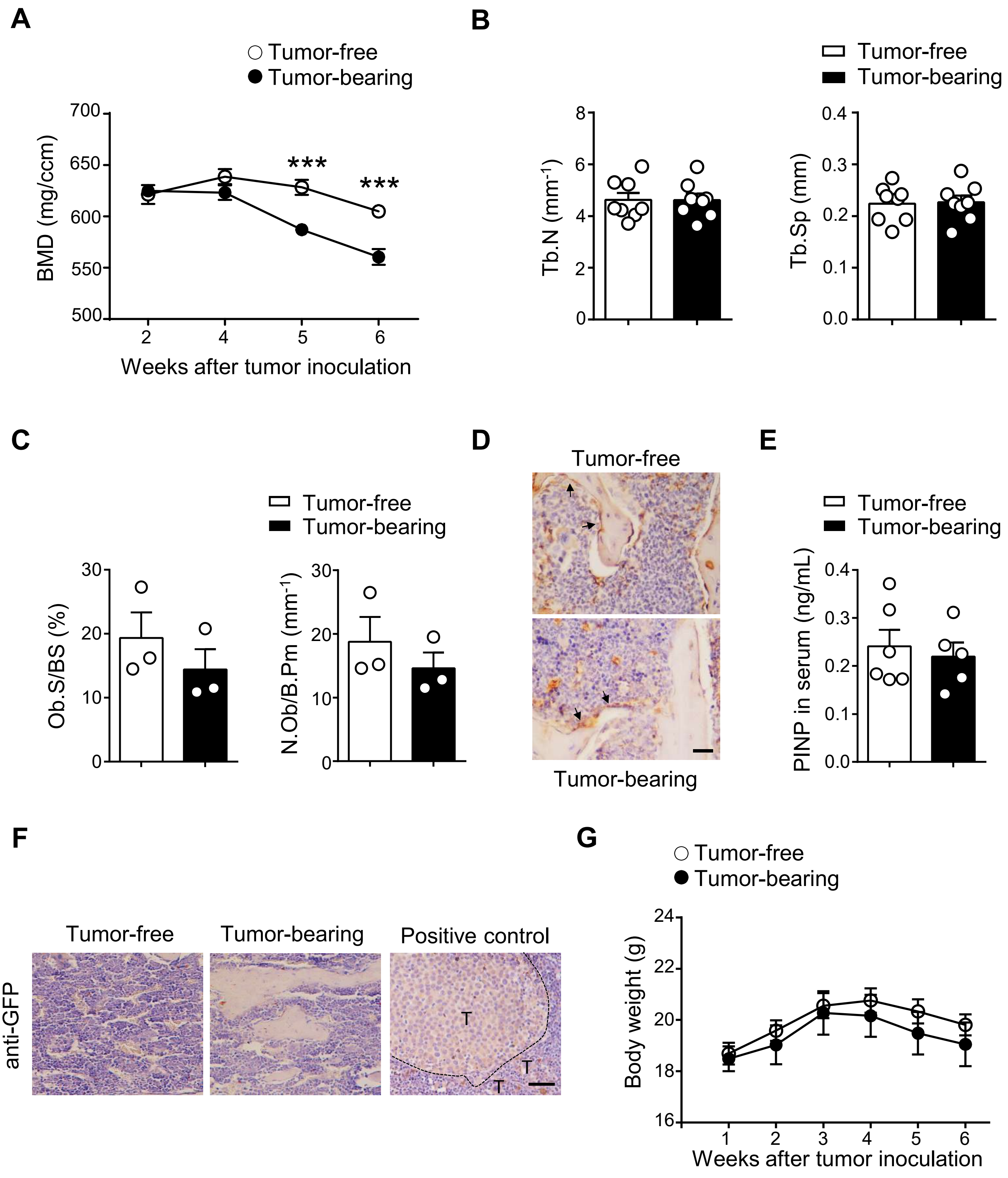

# Yuan et al Supplementary Figure 2

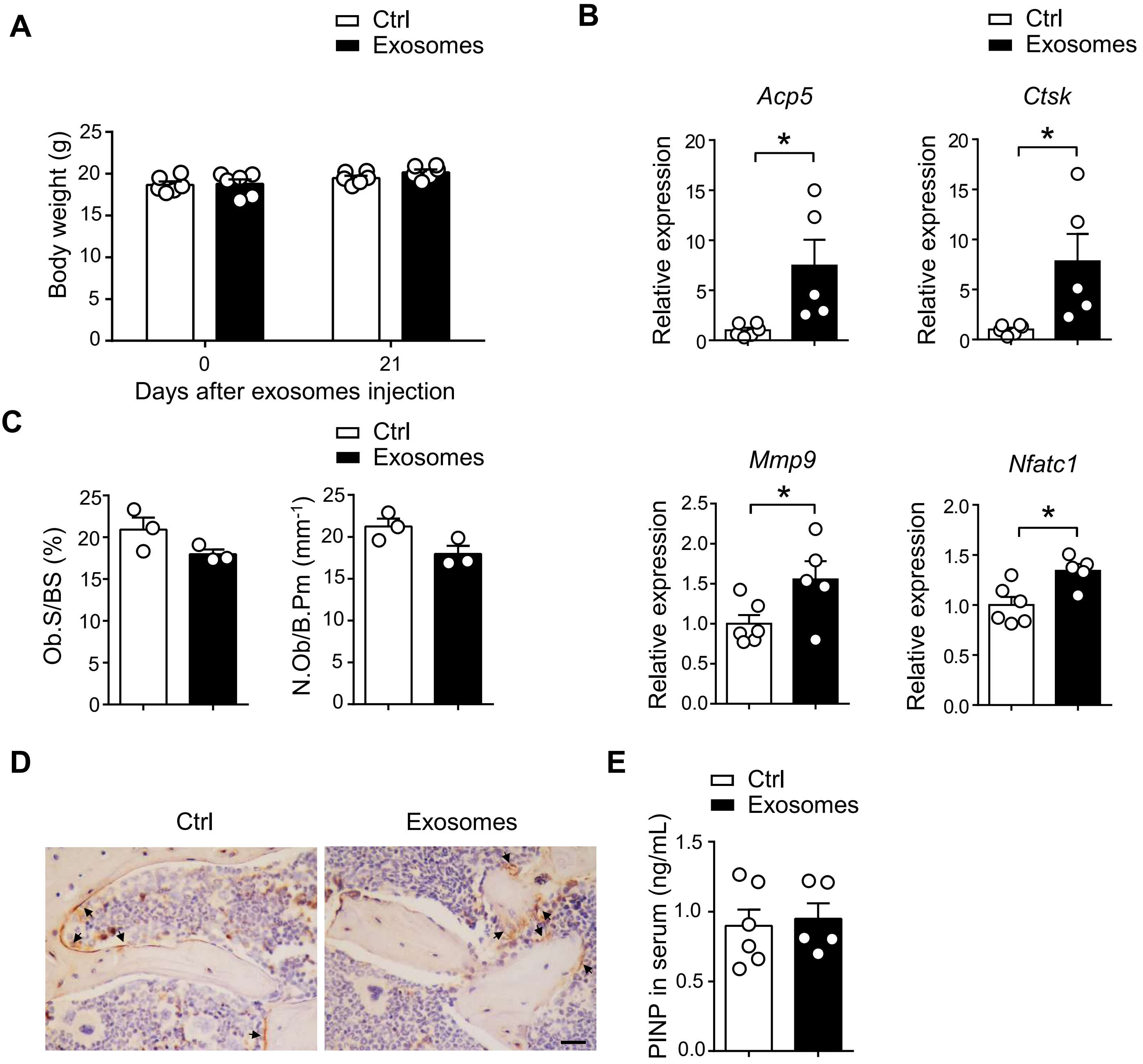

# Yuan et al Supplementary Figure 3

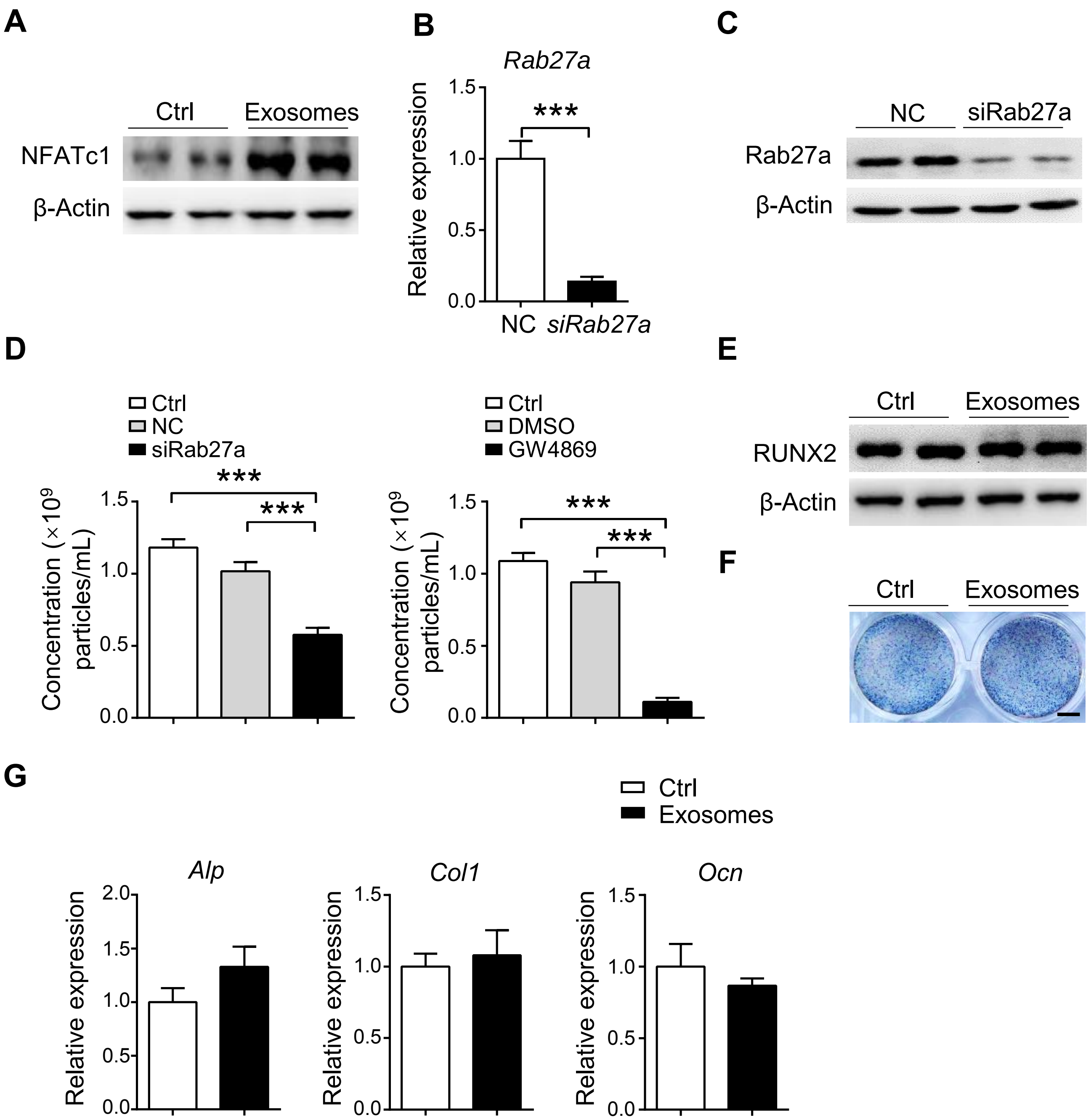

# Yuan et al Supplementary Figure 4

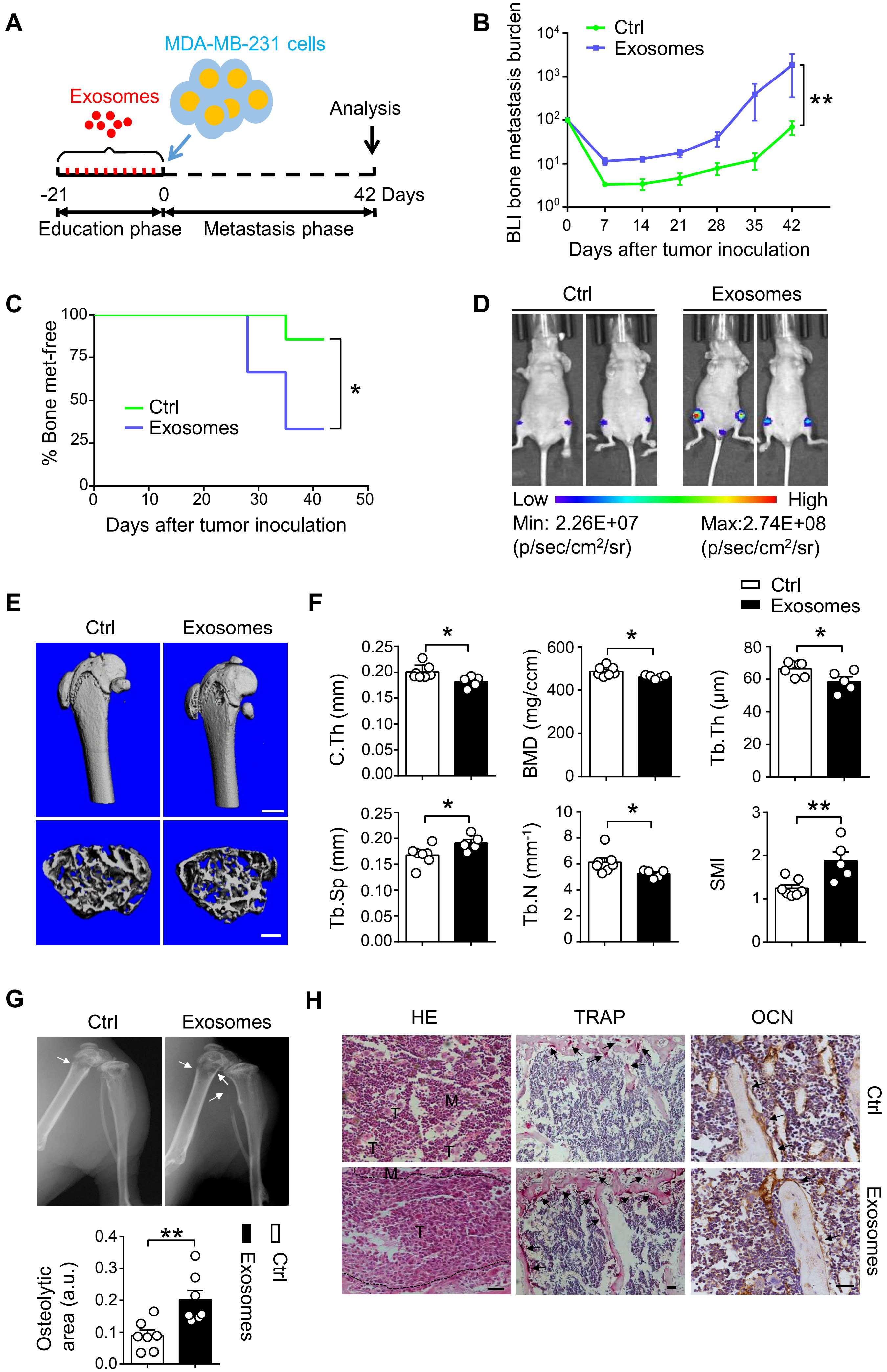

# Yuan et al Supplementary Figure 5

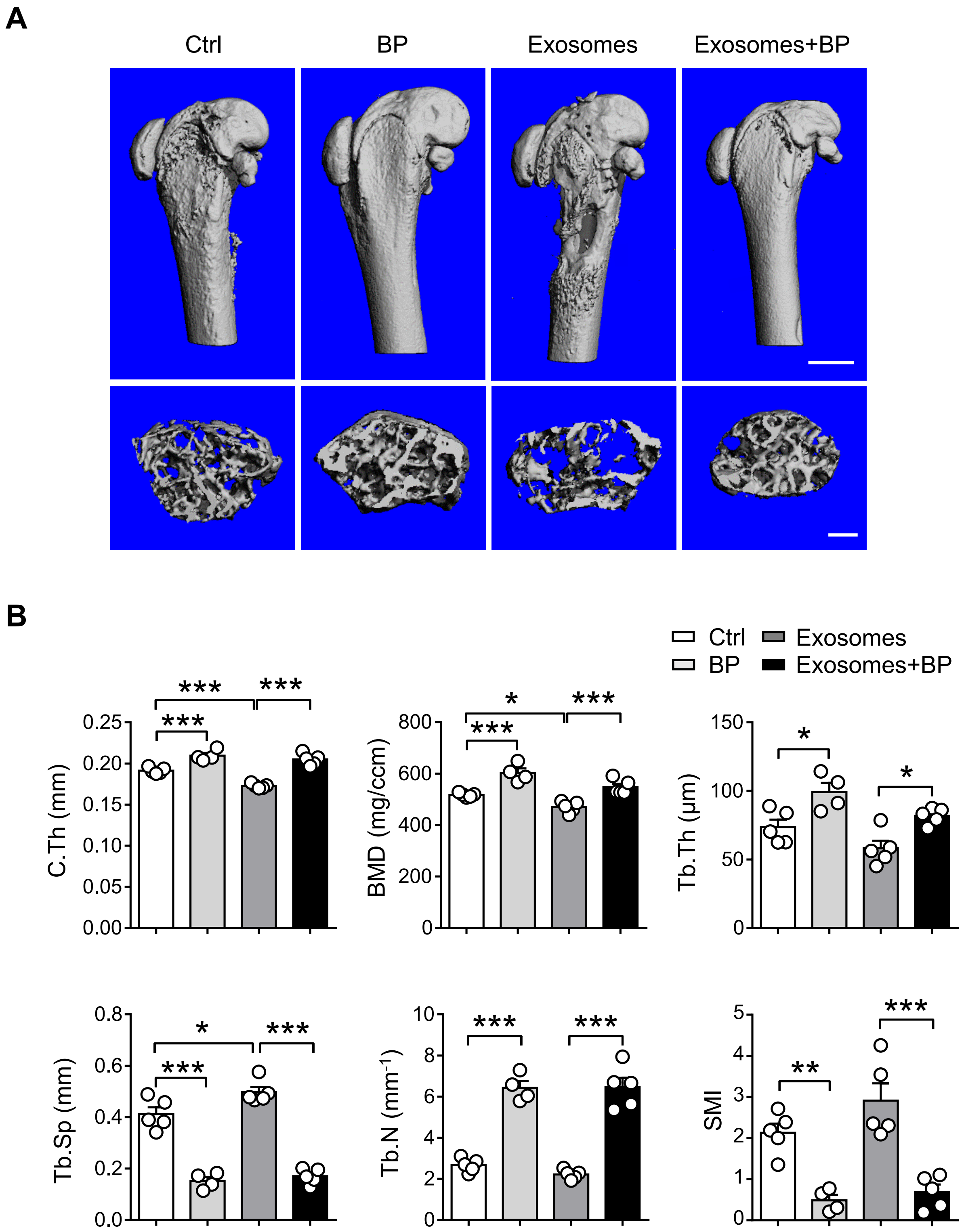

# Yuan et al Supplementary Figure 6

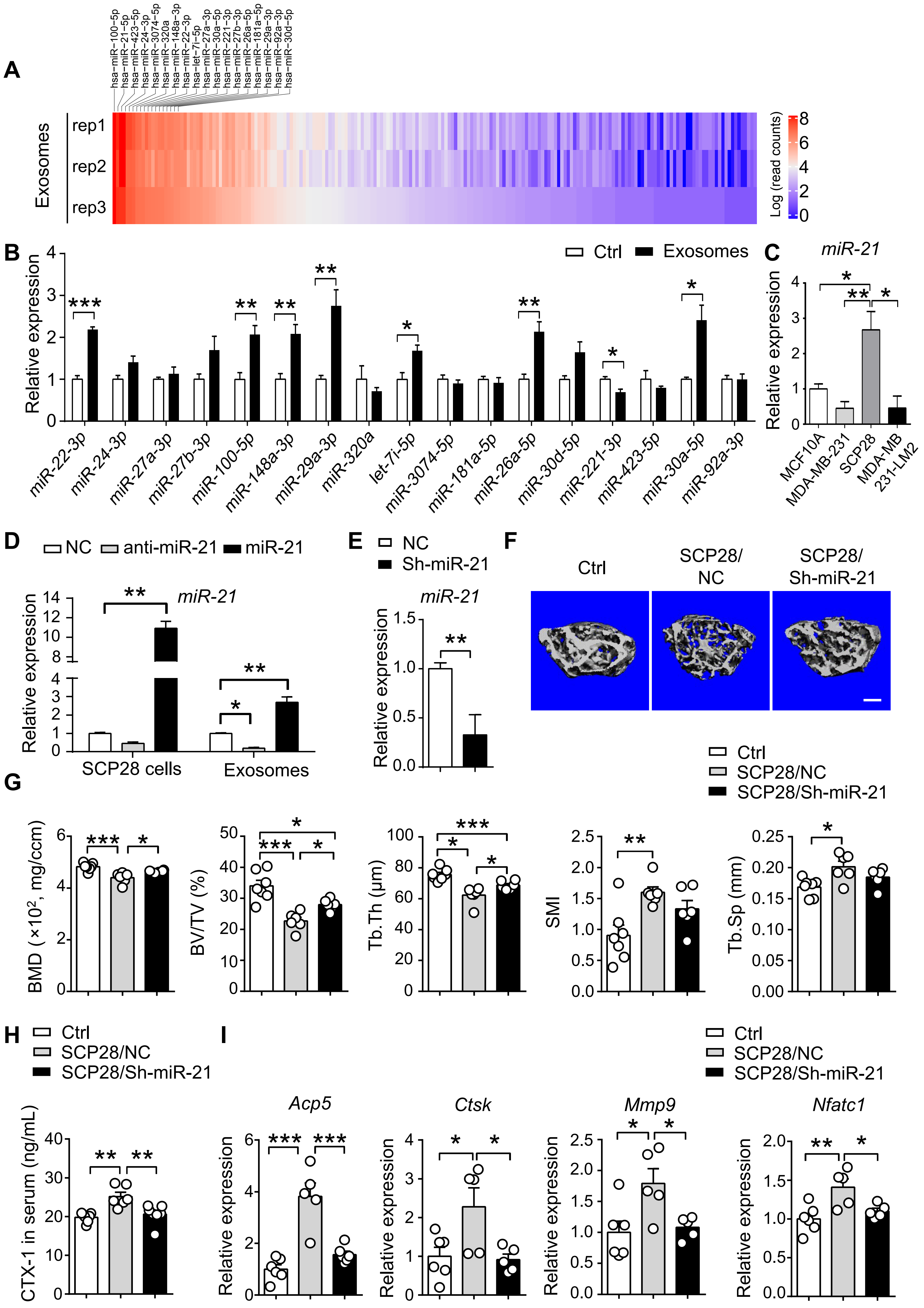

**Table S1. The primer list for qRT-PCR**

| Name | sense primer (5'-3') | anti-sense primer (5'-3') |
|---|---|---|
| mmu-*Gapdh* | ATGGTGAAGGTCGGTGTGAA | GTCGTTGATGGCAACAATCTCC |
| mmu-*Nfatc1* | ACGCTACAGCTGTTCATTGG | CTTTGGTGTTGGACAGGATG |
| mmu-*Acp5* | GCGACCATTGTTAGCCACATACG | CGTTGATGTCGCACAGAGGGAT |
| mmu-*Mmp9* | GCGGCCCTCAAAGATGAACGG | GCTGACTACGATAAGGACGGCA |
| mmu-*Ctsk* | GCGTTGTTCTTATTCCGAGC | CAGCAGAGGTGTGTACTATG |
| mmu-*Alp* | ATCTTTGGTCTGGCTCCCATG | TTTCCCGTTCACCGTCCAC |
| mmu-*Ocn* | CCAAGCAGGAGGGCAATA | TCGTCACAAGCAGGGTCA |
| mmu-*Col1α1* | GGGACCAGGAGGACCAGGAAGT | GGAGGGCGAGTGCTGTGCTTT |
| mmu-*Pdcd4* | AGCGGTTAGAAGTGGAGTTGCTGT | ACAAGGTGATTGACAGGCTGTTGC |
| hsa-*Gapdh* | GGAGCGAGATCCCTCCAAAAT | GGCTGTTGTCATACTTCTCATGG |
| hsa-*Rab27a* | ACAACAGTGGGCATTGATTTCA | AAGCTACGAAACCTCTCCTGC |
| hsa-*Pre-miR-21* | CTTTAGGAGCATTATGAGC | ACTATCCCCATTTCTCCA |
| hsa-*miR-21-5p* | TAGCTTATCAGACTGATGTTGA | GAATCGAGCACCAGTTACGC |
| hsa-*miR-22-3p* | AAGCTGCCAGTTGAAGAACTGT | GAATCGAGCACCAGTTACGC |
| hsa-*miR-24-3p* | TGGCTCAGTTCAGCAGGAACAG | GAATCGAGCACCAGTTACGC |
| hsa-*miR-27a-3p* | TTCACAGTGGCTAAGTTCCGC | GAATCGAGCACCAGTTACGC |
| hsa-*miR-27b-3p* | TTCACAGTGGCTAAGTTCTGC | GAATCGAGCACCAGTTACGC |
| hsa-*miR-100-5p* | AACCCGTAGATCCGAACTTGTG | GAATCGAGCACCAGTTACGC |
| hsa-*miR-148a-3p* | TCAGTGCACTACAGAACTTTGT | GAATCGAGCACCAGTTACGC |
| hsa-*miR-29a-3p* | TAGCACCATCTGAAATCGGTTA | GAATCGAGCACCAGTTACGC |
| hsa-*miR-320a* | AAAAGCTGGGTTGAGAGGGCGA | GAATCGAGCACCAGTTACGC |
| hsa-*let-7i-5p* | TGAGGTAGTAGTTTGTGCTGTT | GAATCGAGCACCAGTTACGC |
| hsa-*miR-3074-5p* | GTTCCTGCTGAACTGAGCCAG | GAATCGAGCACCAGTTACGC |
| hsa-*miR-181a-5p* | AACATTCAACGCTGTCGGTGAGT | GAATCGAGCACCAGTTACGC |
| hsa-*miR-26a-5p* | TCAAGTAATCCAGGATAGGCT | GAATCGAGCACCAGTTACGC |
| hsa-*miR-30d-5p* | TGTAAACATCCCCGACTGGAAG | GAATCGAGCACCAGTTACGC |
| hsa-*miR-221-3p* | AGCTACATTGTCTGCTGGGTTTC | GAATCGAGCACCAGTTACGC |
| hsa-*miR-423-5p* | TGAGGGGCAGAGAGCGAGACTTT | GAATCGAGCACCAGTTACGC |
| hsa-*miR-30a-5p* | TGTAAACATCCTCGACTGGAAG | GAATCGAGCACCAGTTACGC |
| hsa-*miR-92a-3p* | TATTGCACTTGTCCCGGCCTGT | GAATCGAGCACCAGTTACGC |
| hsa-*miR-16* | TAGCAGCACGTAAATATTGGCG | GAATCGAGCACCAGTTACGC |
| cel-*miR-39* | GGCGCTACCTGTATCAATGG | GTGGTCAGCCAACTCGTCA |
| *U6* | CGCTTCGGCAGCACATATA | TTCACGAATTTGCGTGTCAT |
